# A high-risk sepsis subtype identified by regulated cell death signatures and MAD2L2 validation

**DOI:** 10.3389/fimmu.2026.1846661

**Published:** 2026-08-27

**Authors:** Lifang Luo, Dong Cao, Zhaoxia Liao, Chongyang Lin, Anhui Wang, Le Zeng, Jie Yang, Wen Song, Yiyu Deng

**Affiliations:** 1 School of Medicine, South China University of Technology, Guangzhou, Guangdong, China; 2 Department of Critical Care Medicine, Guangdong Provincial People’s Hospital, Guangdong Academy of Medical Sciences, Southern Medical University, Guangzhou, Guangdong, China; 3 Department of Anesthesiology, Sun Yat-sen Memorial Hospital, Sun Yat-sen University, Guangzhou, China; 4 Southern Medical University, Guangzhou, Guangdong, China

**Keywords:** machine-learning, MAD2L2, molecular endotyping, RCD.score, regulated cell death (RCD), sepsis

## Abstract

Regulated cell death (RCD) is closely involved in immune dysregulation and organ injury during sepsis, yet the coordinated transcriptional features of multiple RCD modalities and their relevance to sepsis heterogeneity remain insufficiently characterized. Using the whole-blood transcriptomic cohort GSE65682, we performed consensus clustering of RCD-associated genes and identified four molecular subtypes among 479 patients with sepsis. Cluster 3 showed the poorest 28-day survival (log-rank p = 0.034) and was characterized by a distinct mixed immune transcriptional profile. A sensitivity analysis restricted to apoptosis-, ferroptosis-, necroptosis-, and pyroptosis-associated genes reproduced the primary four-cluster structure and retained the high-risk features of Cluster 3. Integrated differential-expression analysis, weighted gene co-expression network analysis, and complementary machine-learning approaches identified a six-gene signature comprising SMC4, SLC5A6, POLR3GL, MAD2L2, EXOC7, and COG1. The resulting RCD.score closely captured the transcriptional phenotype of Cluster 3. In the external GSE54514 cohort, patients with higher RCD.score values had higher APACHE II scores, and exploratory joint stratification identified a subgroup with both high APACHE II and high RCD.score that had the highest observed mortality. Single-cell analysis localized much of the RCD.score-associated signal to monocyte and dendritic-cell populations. Sample-level analyses showed the clearest sepsis-associated increase in monocytes, with MAD2L2 making the largest positive contribution among the six signature genes. In THP-1 cells, LPS stimulation increased MAD2L2, TNF-α, and IL-1β protein expression, whereas MAD2L2 knockdown reduced these inflammatory proteins and decreased GSDMD-N abundance and caspase-1 processing under both LPS and LPS + ATP conditions. These findings define an RCD-associated high-risk sepsis subtype, provide a cell-type-resolved view of its molecular features, and identify MAD2L2 as a candidate involved in monocyte inflammatory and pyroptosis-associated responses.

## Graphical Abstract

**Figure:**
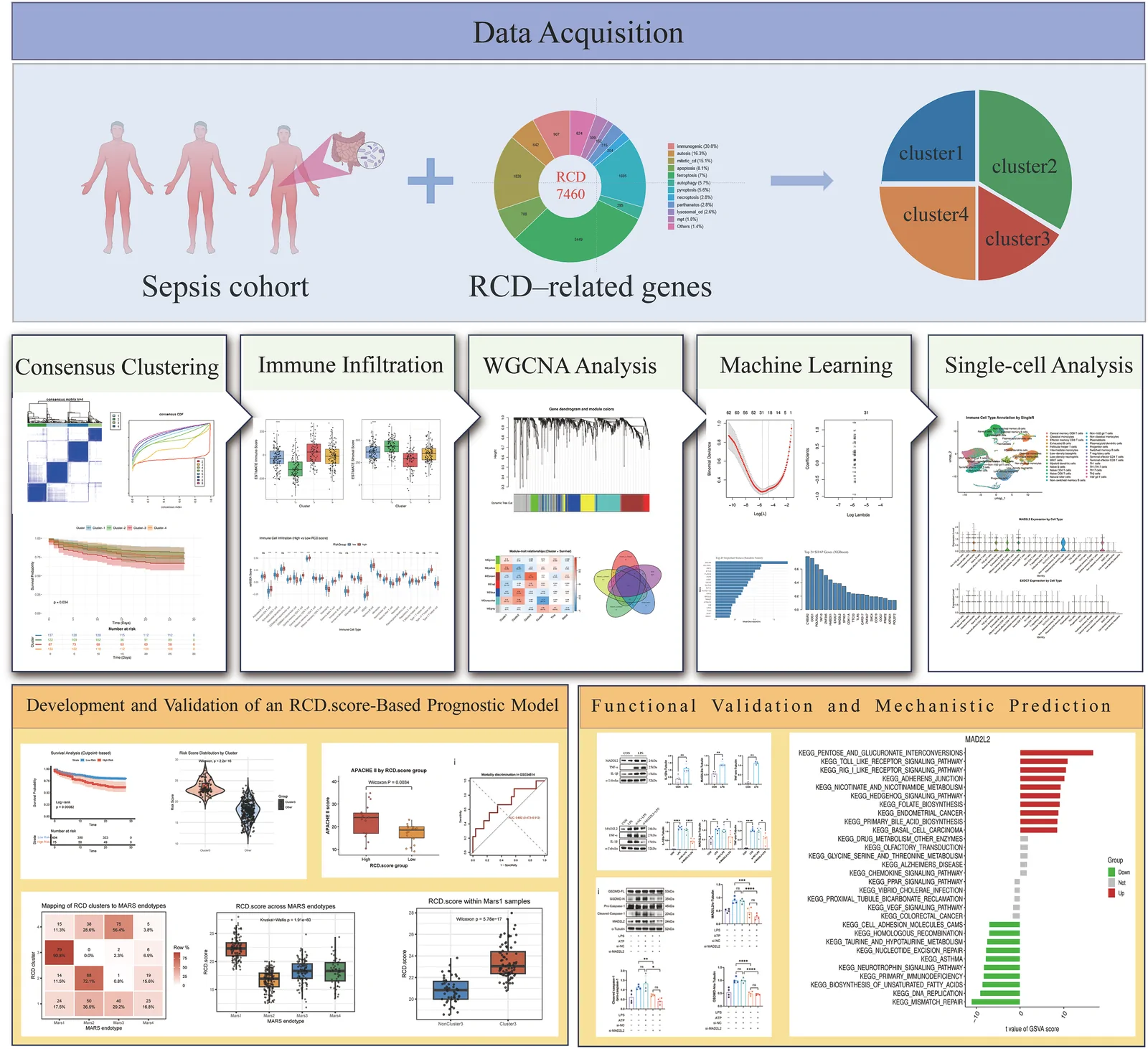
Integration of whole-blood transcriptomic profiles with curated regulated cell-death genes identified four sepsis subtypes, including a high-risk Cluster 3 with the poorest 28-day survival and a mixed immune transcriptional state. The subtype structure was reproduced using a restricted apoptosis-, ferroptosis-, necroptosis-, and pyroptosis-related gene set. A six-gene RCD.score captured the Cluster 3 molecular phenotype, and single-cell analyses localized much of the associated signal to monocyte and dendritic-cell populations. MAD2L2 showed prominent monocyte expression, and its knockdown in THP-1 cells reduced TNF-α and IL-1β expression, GSDMD-N abundance, and caspase-1 processing. These findings support MAD2L2 as a candidate involved in monocyte inflammatory and pyroptosis-associated responses.

## Introduction

Sepsis is a critical clinical illness resulting from a dysregulated host response to infection, often leading to organ damage and a significant risk of mortality. Worldwide, sepsis affects an estimated 49 million people each year and is associated with roughly 11 million deaths, representing nearly one fifth of all deaths globally (1). Despite advancements in antimicrobial therapies and supportive care, the prognosis of sepsis remains highly variable among patients, largely due to its complex pathophysiology and clinical heterogeneity (2). In routine clinical practice, disease severity is commonly evaluated using established diagnostic and prognostic scoring systems, including SOFA and APACHE II (3). However, these scoring systems are predominantly based on physiological and biochemical parameters and fail to reflect the underlying molecular heterogeneity that drives individual differences in disease progression and treatment response (4). Therefore, identifying robust molecular subtypes of sepsis is critical to advancing precision medicine and tailoring therapeutic strategies to specific patient subgroups.

Multiple genetically regulated cell-death pathways—most notably apoptosis, pyroptosis, ferroptosis, and necroptosis—are collectively classified under regulated cell death (RCD) (5). Recent work suggests that dysregulated RCD pathways are deeply involved in shaping immune dysfunction and organ injury in sepsis (6, 7). For instance, Inflammasome-driven pyroptosis activates caspase-1, induces gasdermin D–dependent membrane pore formation, and enables the release of IL-1β and IL-18 (8). Dysregulated pyroptotic activity can fuel excessive cytokine production, accelerating tissue injury and organ failure in the septic setting (9). Iron-dependent lipid peroxidation–mediated cell death, termed ferroptosis, has been increasingly implicated in sepsis-related liver and kidney injury. For example, dysregulation of the NFIL3–ACSL4 signaling axis has been shown to enhance ferroptosis and inflammatory activation in renal tubular epithelial cells during sepsis-associated acute kidney injury, ultimately worsening renal damage (10). While several studies have examined the mechanistic relevance of individual RCD pathways in sepsis, the coordinated transcriptional patterns of multiple RCD modalities and their relationship to sepsis heterogeneity remain incompletely characterized (11, 12). It also remains unclear whether an RCD-anchored framework can identify clinically relevant molecular subtypes and reveal the cellular programs associated with high-risk disease.

The integration of high-throughput transcriptomic profiling with publicly available large-scale datasets has enabled systematic molecular stratification and outcome prediction. Bioinformatics tools such as Weighted gene co-expression network analysis (WGCNA) and unsupervised clustering techniques have been widely applied to identify disease subtypes in cancer, autoimmune disorders, and acute inflammatory conditions (13). In sepsis, transcriptomic approaches are increasingly being adopted to identify molecular subtypes associated with divergent immune responses and outcomes (14). In parallel, machine learning approaches such as LASSO, random forest, and XGBoost have proven effective for feature selection in high-dimensional data and for constructing robust risk scores, with successful applications in prognostic prediction across a range of diseases (15–17). Therefore, integrating bioinformatics and machine learning strategies may offer promising tools for personalized risk stratification and targeted interventions in sepsis. However, the integration of these computational approaches with RCD-associated transcriptional features for molecular subtyping and cell-type-resolved characterization in sepsis remains limited.

To address these gaps, we developed an RCD-anchored framework to characterize molecular heterogeneity in sepsis and identify a clinically relevant high-risk subtype. Using publicly available peripheral-blood transcriptomic cohorts, we applied consensus clustering and WGCNA to define RCD-associated molecular subtypes and identify subtype-related co-expression programs. We then integrated LASSO, random forest, and XGBoost feature selection to derive a parsimonious six-gene RCD.score that captured the transcriptional characteristics of the high-risk subtype. We further characterized subtype- and score-associated immune transcriptional patterns using ESTIMATE-derived scores and ssGSEA, mapped their cellular distribution using single-cell RNA sequencing, and performed complementary validation in THP-1 cells. This framework provides an RCD-centered approach for molecular characterization of sepsis heterogeneity and for prioritizing candidate cellular and molecular features for further investigation.

## Methods

### Data extraction and processing

Gene-expression and clinical data were obtained from the Gene Expression Omnibus. GSE65682, comprising peripheral-blood transcriptomic profiles and 28-day survival information from patients with sepsis, was used for molecular subtyping and RCD.score development (18). Expression data were processed using GEOquery and limma (19, 20). Probes were mapped to gene symbols, and when multiple probes represented the same gene, the probe with the highest mean expression was retained.

GSE54514 was used for external clinical contextualization. Analyses were restricted to Day 1 sepsis samples to avoid repeated observations from serial sampling. The fixed six-gene composition and coefficients derived from GSE65682 were applied without refitting the RCD.score; the cohort median was used only for exploratory high- and low-RCD.score stratification. This cohort was used to assess the relationship of RCD.score with APACHE II and survival status, perform exploratory joint stratification, and evaluate incremental value beyond APACHE II using DeLong’s test, continuous net reclassification improvement, and integrated discrimination improvement. SOFA was not available in the deposited clinical annotations and was therefore not evaluated. Detailed procedures are provided in the **Supplementary Methods**.

### RCD gene compendium and consensus clustering

The primary RCD gene compendium was adopted from a previously published pan-cancer study that assembled 18 modality-specific RCD signatures from public databases and published literature and merged multiple source lists within each modality (21). No genes were added or removed in the present study on the basis of sepsis-associated expression, differential expression, molecular-subtype assignment, or clinical outcome. A gene was included if it was assigned to at least one RCD modality in the source compendium. Duplicate gene symbols were collapsed to a single entry for clustering, while all original gene–modality assignments were retained in **Supplementary Table 1**. This yielded 7, 460 unique RCD-associated genes, of which 4, 929 were represented in the processed GSE65682 expression matrix and used for consensus clustering after gene-wise z-score standardization.

Consensus clustering was performed using ConsensusClusterPlus for candidate solutions from k = 2 to k = 9, with 100 resampling iterations, pItem = 0.8, pFeature = 1.0, k-means clustering, and Euclidean distance. The four-cluster solution was selected by jointly considering consensus matrices, cumulative distribution function curves, changes in the area under the cumulative distribution function, cluster separation, and cluster-size distribution (22).

### Restricted RCD gene-set sensitivity analysis

As a sensitivity analysis, consensus clustering was repeated using a restricted gene set comprising apoptosis-, ferroptosis-, necroptosis-, and pyroptosis-associated genes. The prespecified four-cluster solution was compared with the primary classification using multiple concordance measures, and its association with 28-day survival was assessed. Detailed gene-set construction, clustering parameters, and concordance analyses are provided in the **Supplementary Methods**.

### Comparison with established MARS and SRS transcriptomic endotypes

The RCD-defined clusters were compared with published MARS endotypes and the Davenport Sepsis Response Signature classification in GSE65682 (23, 24). Published MARS annotations were used directly, whereas formal SRS groups and quantitative SRS scores were assigned using SepstratifieR and the seven-gene Davenport signature. Concordance and discordance between classification systems were assessed using contingency tables, Cramér’s V, subtype-enrichment tests, comparisons of RCD.score across established endotypes, and within-Mars1 analyses. Sequential Cox models were used to evaluate whether RCD.score retained prognostic information beyond MARS endotyping. Detailed assignment parameters, outlier handling, and sensitivity analyses are described in the **Supplementary Methods**.

### Kaplan–Meier survival analysis

Twenty-eight-day survival was compared across molecular subtypes and RCD.score groups using Kaplan–Meier curves and log-rank tests (25). Cox proportional-hazards models were used to estimate hazard ratios, and proportional-hazards assumptions were evaluated using Schoenfeld residuals.

### Differential expression and functional enrichment analyses

Differential expression was analysed using limma with empirical Bayes moderation. Genes with |log_2_ fold change| > 1 and a Benjamini–Hochberg-adjusted FDR < 0.05 were considered differentially expressed. Gene Ontology and KEGG enrichment analyses were performed using clusterProfiler and org.Hs.eg.db, and significant terms were summarized according to adjusted *p* values (26).

### Immune-related transcriptomic analyses

ESTIMATE-derived Immune, Stromal, and ESTIMATE Scores were calculated from normalized whole-blood expression data (27). Because ESTIMATE was developed for tissue transcriptomes, these scores were interpreted as descriptive expression-derived indices rather than direct estimates of cell abundance or tissue structure. Relative enrichment of 28 immune-cell-associated signatures was quantified using ssGSEA implemented in GSVA (**Supplementary Table 3**) (28). Differences across subtypes were assessed using Kruskal–Wallis tests followed by Dunn’s comparisons with Benjamini–Hochberg correction.

### Weighted gene co-expression network analysis

Genes with a standard deviation >0.7 were retained for weighted gene co-expression network analysis (13). Sample and gene quality were assessed before construction of an unsigned adjacency matrix and topological overlap matrix. A soft-thresholding power of 10 was selected based on scale-free topology and connectivity. Modules were identified by dynamic tree cutting and merged at an eigengene dissimilarity threshold of 0.25. Module eigengenes were correlated with clinical traits, and genes with |module membership| >0.8 and |gene significance| >0.5 were considered candidate hub genes.

### Candidate-gene prioritization and RCD.score construction

Candidate genes were obtained by intersecting Cluster-3-associated differentially expressed genes, genes from the key WGCNA module, and the curated RCD gene set. Three complementary machine-learning approaches—LASSO, random forest, and XGBoost—were then applied to distinguish Cluster 3 from the other subtypes (29–31). Genes retained by LASSO and ranked among the top 30 features in both random forest and XGBoost were intersected to define the final signature. The RCD.score was calculated as the weighted sum of the expression levels of the selected genes using the corresponding LASSO coefficients. Complete feature-importance rankings and LASSO coefficients are provided in **Supplementary Tables 9–S11**.

### Prognostic evaluation and internal validation of the RCD.score

The six-gene RCD.score was calculated using coefficients derived entirely from GSE65682 and was standardized for Cox analyses so that hazard ratios represented the effect per 1-SD increase. Sequential models included RCD.score alone and models adjusted for age, sex, and MARS endotype. Model fit and discrimination were assessed using likelihood-ratio tests, Akaike information criterion, and Harrell’s C-index. Potential optimism in the feature-selection and score-construction stage was evaluated using 1, 000 patient-level bootstrap resamples beginning from the predefined 122-gene candidate set. In each resample, feature selection and RCD.score construction were repeated, and optimism-corrected AUCs were estimated separately for discrimination of Cluster 3 and 28-day mortality. Further details are provided in the **Supplementary Methods**.

### Single-cell processing and cell-type-resolved analysis of the RCD.score

Single-cell RNA-sequencing data from GSE167363, comprising two healthy control samples and paired baseline and 6-h samples from five patients with sepsis, were obtained from the study by Qiu et al (32). Data were processed using Seurat (33). After quality control, cells were normalized, dimensionally reduced, clustered, and annotated using SingleR with the Monaco Immune reference (34, 35). Per-cell RCD.score values were calculated from the six signature genes using the fixed model coefficients and were used to visualize the cellular distribution of the bulk-derived signature.

For the sample-level comparison, two healthy controls and five baseline sepsis samples were retained. Mean RCD.score and gene-specific weighted contributions were summarized within each sample and major immune-cell population. To provide a complementary genome-wide, cell-type-specific analysis not restricted to the six-gene score definition, raw counts were aggregated by biological sample and cell type for pseudobulk differential expression analysis using edgeR. Additional details of single-cell preprocessing, score calculations, cell-number criteria, and pseudobulk procedures are provided in the **Supplementary Methods**.

### THP-1 cell culture and differentiation

The human monocytic cell line THP-1 (RRID: CVCL_0006) was cultured in RPMI-1640 medium supplemented with 10% fetal bovine serum, 0.05 mM β-mercaptoethanol, and 1% penicillin–streptomycin at 37 °C in a humidified atmosphere containing 5% CO_2_. For experiments, THP-1 cells were seeded and treated with phorbol 12-myristate 13-acetate (PMA, 50 nM) for 24 h to induce adherence. Unless otherwise stated, subsequent treatments and transfections were performed in PMA-adherent cells.

### LPS stimulation, ATP treatment, and siRNA transfection

To induce an inflammatory response, PMA-adherent THP-1 cells were stimulated with lipopolysaccharide (LPS, 1 μg/mL; Sigma-Aldrich) for 6 h. Gene-specific siRNA targeting MAD2L2 (si-MAD2L2) and a non-targeting negative-control siRNA (si-NC) were designed and synthesized by RiboBio (Guangzhou, China); the sequences are provided in **Supplementary Table 12**. Cells at 40–60% confluence were transfected with 50 nM siRNA using the riboFECT™ CP Transfection Kit according to the manufacturer’s instructions. Briefly, siRNA and transfection reagent were premixed in serum- and antibiotic-free medium for 10–15 min at room temperature, added dropwise to the cells, and replaced with complete medium after 6 h. Three experimental comparisons were performed. First, untreated and LPS-stimulated cells were compared to evaluate the effects of LPS on MAD2L2, TNF-α, and IL-1β expression. Second, untreated control, LPS, LPS + si-NC, and LPS + si-MAD2L2 groups were used to evaluate the effects of MAD2L2 knockdown on inflammatory-protein expression. Third, untreated control, LPS + si-NC, LPS + ATP + si-NC, LPS + si-MAD2L2, and LPS + ATP + si-MAD2L2 groups were used to examine GSDMD and caspase-1 processing. In the ATP-containing groups, ATP (5 mM; A9130, Solarbio, Beijing, China) was added during the final 1 h of the 6-h LPS stimulation. siRNA-transfected cells were stimulated 48 h after transfection, and all cells were collected immediately after treatment for immunoblot analysis.

### Western blotting

Cell lysates were prepared in RIPA buffer supplemented with protease inhibitors. Protein concentrations were determined using a BCA assay, and 30 μg of total protein per sample was separated by SDS–PAGE and transferred to PVDF membranes. PVDF membranes were blocked with 5% non-fat milk and incubated overnight at 4 °C with primary antibodies against MAD2L2 (1:1000, 98423S, Cell Signaling Technology, USA), IL-1β (1:1000, 26048-1-AP, Proteintech, China), TNF-α (1:1000, 17590-1-AP, Proteintech, China), total and cleaved caspase-1 (1:1000, P79884R2, Abmart, China), GSDMD and cleaved N-terminal GSDMD (1:1000, PU224937, Abmart, China), and α-tubulin (1:10, 000, 11224-1-AP, Proteintech, China). Following TBST washes, membranes were incubated with horseradish peroxidase-conjugated anti-rabbit or anti-mouse secondary antibodies (1:5000, 7074S or 7076S, Cell Signaling Technology, USA) for 1 h at room temperature. Immunoreactive signals were detected using enhanced chemiluminescence reagent (BL520A, Biosharp, China), captured using a Bio-Rad ChemiDoc imaging system, and quantified in ImageJ. MAD2L2 and GSDMD-N band intensities were normalized to α-tubulin. Caspase-1 processing was quantified as the ratio of cleaved caspase-1 to pro-caspase-1.

### MAD2L2-associated pathway analysis

GSVA was used to compare KEGG pathway-enrichment scores between samples with high and low MAD2L2 expression. Differences were evaluated using limma and summarized using moderated t statistics.

### Statistical analysis

Continuous variables were compared using Student’s t-test, one-way analysis of variance, Wilcoxon rank-sum test, or Kruskal–Wallis test, as appropriate. Categorical variables were compared using the χ² test or Fisher’s exact test. Survival analyses were performed using Kaplan–Meier curves, log-rank tests, and Cox proportional-hazards models. Receiver operating characteristic curves and 95% confidence intervals were used to assess discrimination. For transcriptomic and other high-dimensional analyses, multiple comparisons were corrected using the Benjamini–Hochberg method.

Immunoblot data are presented as mean ± SD from independent biological experiments. For immunoblot experiments involving more than two groups, homogeneity of variance was assessed separately for each protein endpoint. Data satisfying the equal-variance assumption were analysed using ordinary one-way ANOVA followed by Bonferroni’s multiple-comparisons test. When the equal-variance assumption was not met, Brown–Forsythe and Welch ANOVA tests were used, followed by Dunnett’s T3 multiple-comparisons test. Only prespecified pairwise comparisons were performed. All tests were two-sided, and *p* or FDR values below 0.05 were considered statistically significant. Transcriptomic and computational analyses were performed in R version 4.4.2, and immunoblot analyses were performed using GraphPad Prism 10.6.0.

## Results

### RCD-based molecular classification reveals a transcriptionally distinct high-risk sepsis subtype

**Figure 1:**
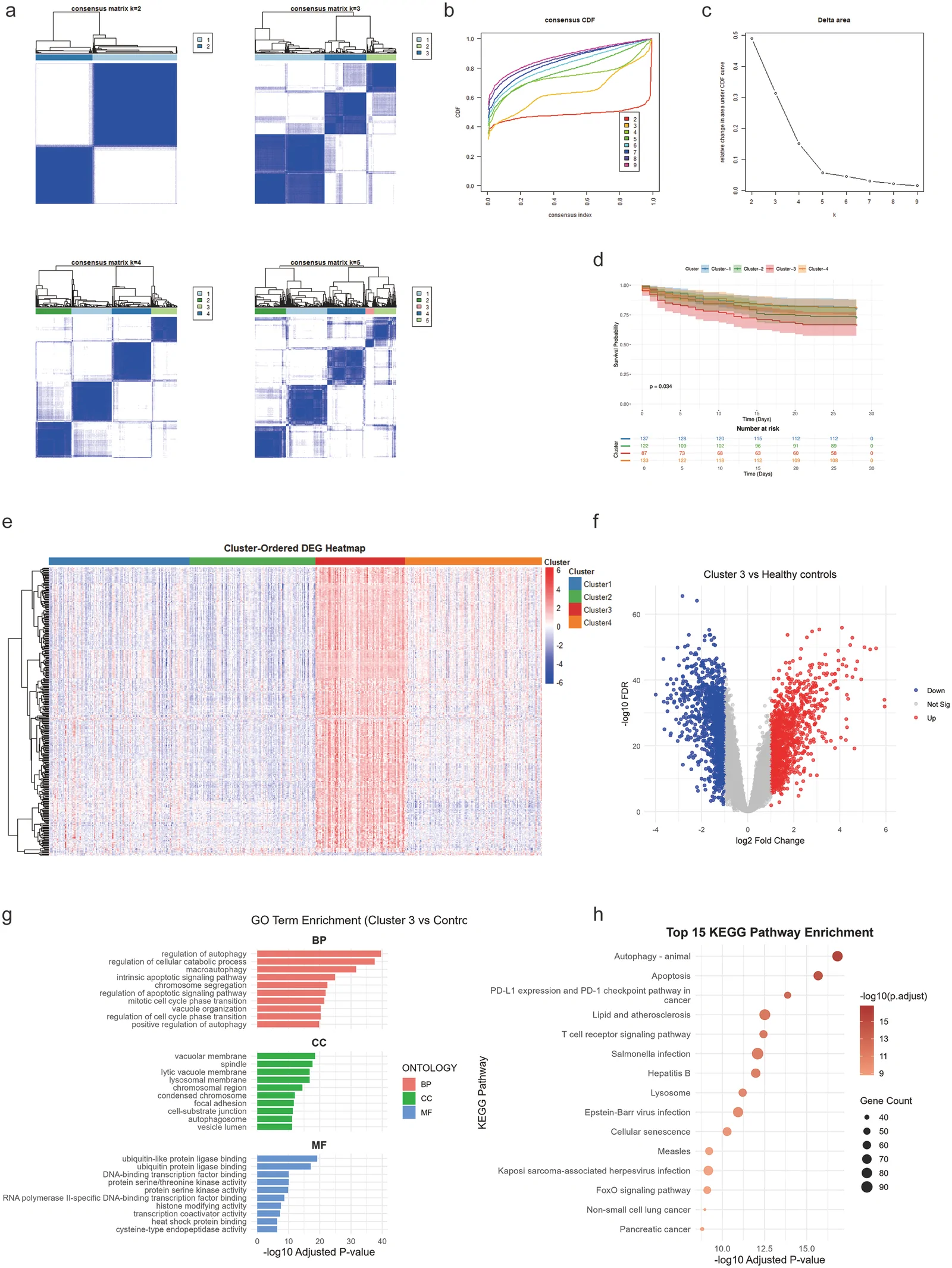
RCD-based molecular classification of sepsis. **(a)** Consensus matrices for candidate cluster solutions from k = 2 to k = 5 based on RCD-associated genes in GSE65682. **(b)** Consensus cumulative distribution function curves for candidate solutions from k = 2 to k = 9. **(c)** Relative change in the area under the cumulative distribution function curve across candidate cluster numbers. A four-cluster solution was used in subsequent analyses. **(d)** Kaplan–Meier curves of 28-day survival across the four RCD-defined subtypes. **(e)** Heatmap of differentially expressed genes across the four RCD-defined subtypes. Expression values are shown as row z-scores, and samples are ordered by subtype. **(f)** Volcano plot of differential expression between Cluster 3 and healthy controls. **(g)** GO enrichment (BP/CC/MF) of Cluster-3–specific DEGs (adjusted *p* values). **(h)** KEGG pathway enrichment (top 15), dot size = gene count, color = adjusted *p*. RCD, regulated cell death; CDF, cumulative distribution function; DEG, differentially expressed gene; FDR, false discovery rate; GO, Gene Ontology; KEGG, Kyoto Encyclopedia of Genes and Genomes.

Of the 7, 460 unique genes included in the published RCD compendium, 4, 929 were represented in the processed GSE65682 expression matrix and were used for consensus clustering of 479 patients with sepsis. Evaluation of the consensus matrices, cumulative distribution function curves, delta-area plot, cluster separation, and cluster-size distribution supported a four-cluster solution (**Figures 1a–c**). Twenty-eight-day survival differed significantly across the four RCD-defined subtypes, with Cluster 3 showing the poorest survival (log-rank *p* = 0.034; **Figure 1d**).

To assess whether the identified subtype structure was sensitive to the breadth of the RCD compendium, we repeated consensus clustering using a restricted gene set comprising apoptosis-, ferroptosis-, necroptosis-, and pyroptosis-associated genes. After deduplication and platform matching, 1, 616 genes were retained for analysis. The prespecified four-cluster solution showed strong concordance with the primary classification, with an adjusted Rand index of 0.951 and a normalized mutual information of 0.933. Overall, 470 of 479 patients were assigned to the corresponding clusters, and 85 of the 87 patients in primary Cluster 3 remained in the corresponding restricted Cluster 3, yielding a retention rate of 97.7% (**Supplementary Figure 1a**).

Survival remained significantly different across the four restricted-set clusters (overall log-rank *p* = 0.038; **Supplementary Figure 1b**). Restricted Cluster 3 had the highest 28-day mortality, with 28 deaths among 86 patients (32.6%), compared with 27 of 140 patients in Cluster 1 (19.3%), 33 of 118 in Cluster 2 (28.0%), and 26 of 135 in Cluster 4 (19.3%). When compared with the remaining clusters combined, restricted Cluster 3 was associated with a higher 28-day mortality risk (HR 1.62, 95% CI 1.06–2.49; *p* = 0.026). The proportional-hazards assumption was not violated (global p = 0.31, **Supplementary Table 14**).

Differential expression analysis further demonstrated a distinct transcriptional profile in Cluster 3. The DEG heatmap highlighted a characteristic expression pattern that distinguished Cluster 3 from the other RCD-defined subtypes, while comparison with healthy controls revealed broad transcriptional alterations (**Figures 1e, f**). GO enrichment analysis of Cluster-3-associated DEGs highlighted autophagy-related processes, apoptotic signaling, mitotic cell-cycle phase transition, and vacuolar organization (**Figure 1g**). KEGG analysis further identified pathways related to autophagy, apoptosis, PD-L1 expression and PD-1 checkpoint signaling, T-cell receptor signaling, lysosomal function, and cellular senescence (**Figure 1h**).

### Cluster 3 exhibits a distinct immune transcriptional profile with concurrent activation- and regulation-associated signatures

**Figure 2:**
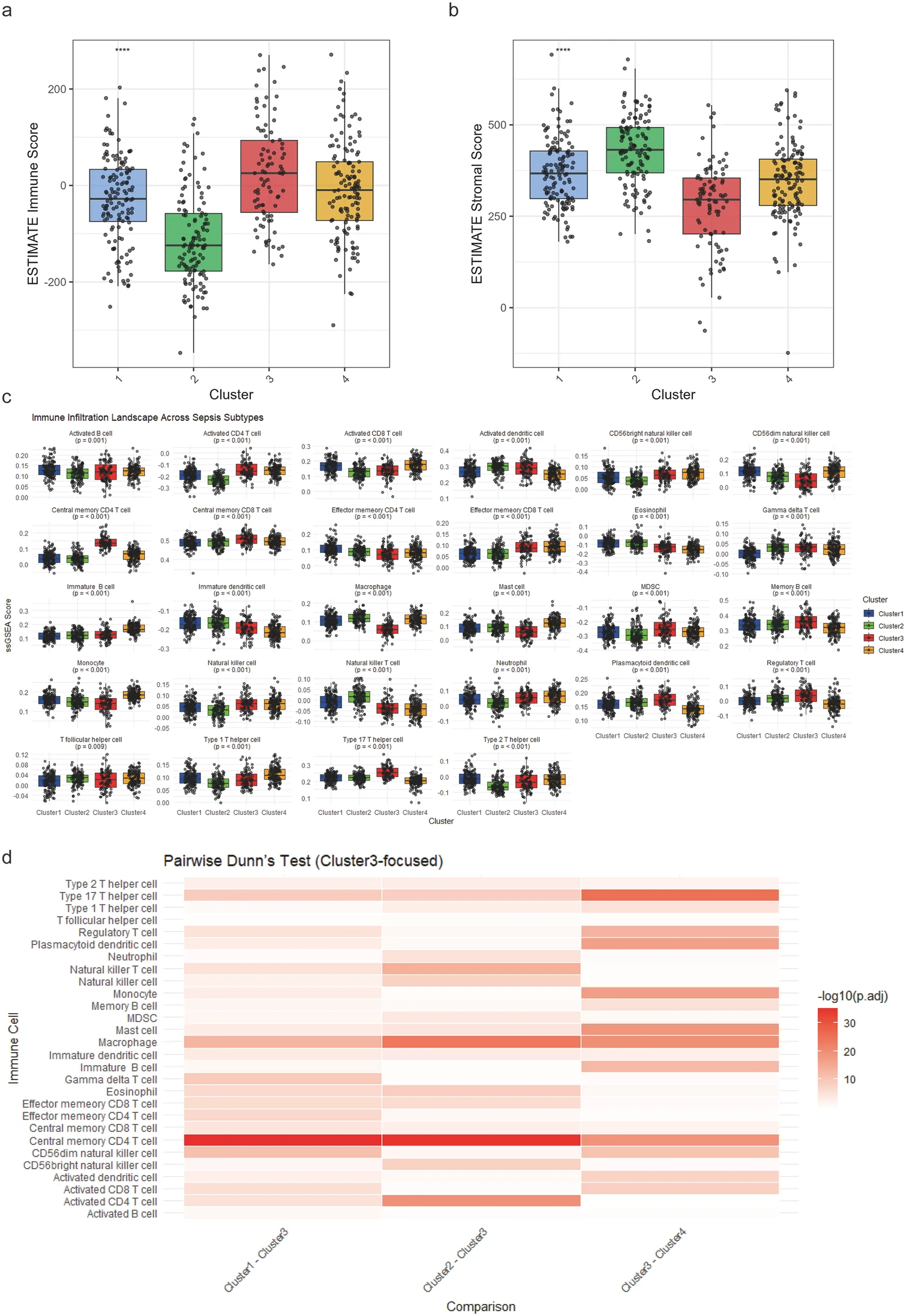
Immune-related transcriptomic features across RCD-defined sepsis subtypes. **(a, b)** ESTIMATE-derived Immune Score **(a)** and Stromal Score **(b)** across the four RCD-defined subtypes in GSE65682. **(c)** ssGSEA enrichment scores for 28 immune-cell-associated signatures across the four RCD-defined subtypes. **(d)** Dunn’s pairwise comparisons of Cluster 3 with the other RCD-defined subtypes for the 28 immune-cell-associated signatures. Columns show Cluster 1 versus Cluster 3, Cluster 2 versus Cluster 3, and Cluster 3 versus Cluster 4. RCD, regulated cell death; ESTIMATE, Estimation of STromal and Immune cells in MAlignant Tumor tissues using Expression data; ssGSEA, single-sample gene set enrichment analysis.

To characterize immune-related transcriptional differences across the RCD-defined subtypes, we first compared ESTIMATE-derived scores. Cluster 3 showed the highest Immune Score and the lowest Stromal Score among the four subtypes (**Figures 2a, b**). Because these scores were derived from whole-blood transcriptomes, they were interpreted as descriptive expression-derived indices rather than direct estimates of immune- or stromal-cell abundance. ssGSEA further revealed a heterogeneous immune-cell-associated signature profile in Cluster 3 (**Figures 2c, d**; **Supplementary Tables 4–S6**). All 28 signatures differed significantly across the four RCD-defined subtypes after multiple-testing correction. Central-memory CD4 T-cell, central-memory CD8 T-cell, and Th17-cell signature scores were higher in Cluster 3 than in all three other subtypes. Cluster 3 also showed higher activated CD4 T-cell, CD56 bright natural killer-cell, effector-memory CD8 T-cell, natural killer-cell, and MDSC scores than Clusters 1 and 2, whereas regulatory T-cell and plasmacytoid dendritic-cell scores were higher than those in Clusters 1 and 4. In contrast, CD56 dim natural killer-cell, macrophage, and mast-cell scores were lower in Cluster 3 than in all other subtypes. These findings indicate a mixed immune transcriptional state rather than uniform enrichment of a single immune-cell program.

### WGCNA identifies a cluster 3-associated co-expression module and candidate genes

**Figure 3:**
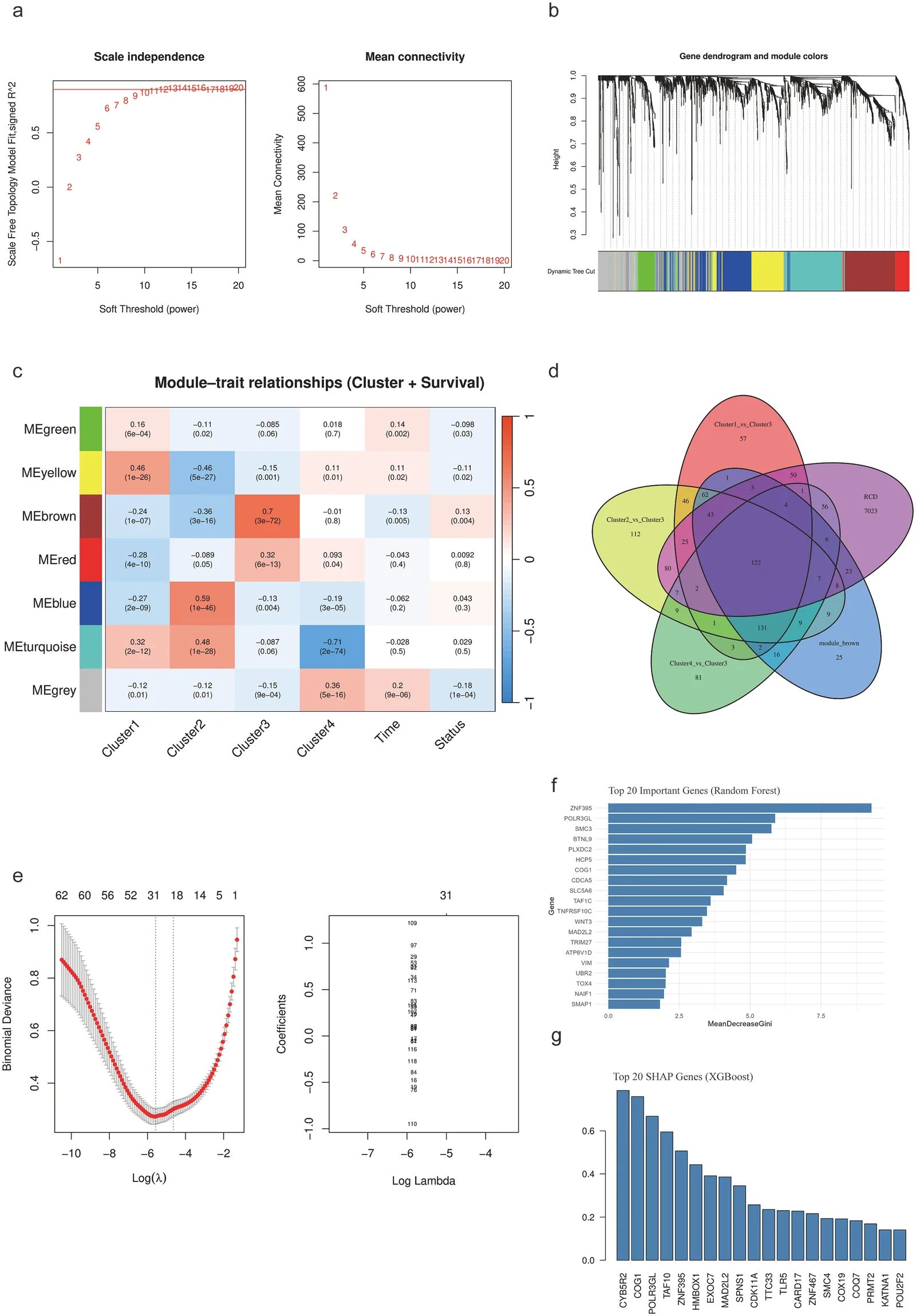
Integrative WGCNA and machine-learning feature selection for candidate-gene prioritization. **(a)** Scale-free topology fit index and mean connectivity across candidate soft-thresholding powers. **(b)** WGCNA gene dendrogram with module colors (dynamic tree cut). **(c)** Module–trait relationships (subtype labels and survival), heat map shows Pearson *r* and *p*. **(d)** Venn diagram showing the intersection of genes in the brown module, the curated RCD gene set, and the differentially expressed genes from the three pairwise comparisons of Cluster 3 with Clusters 1, 2, and 4. The central intersection contained 122 genes. **(e)** LASSO logistic-regression analysis. The left panel shows 10-fold cross-validated binomial deviance across values of λ, and the right panel shows coefficient profiles. **(f)** Random-forest variable-importance ranking (top features by MeanDecreaseGini). **(g)** XGBoost variable-importance ranking based on mean absolute SHAP values. WGCNA, weighted gene co-expression network analysis; RCD, regulated cell death; LASSO, least absolute shrinkage and selection operator; XGBoost, extreme gradient boosting; SHAP, SHapley Additive exPlanations.

To identify co-expression programs associated with the high-risk subtype, we performed weighted gene co-expression network analysis of the sepsis whole-blood transcriptomes. A soft-thresholding power of β = 10 achieved approximate scale-free topology (**Figure 3a**). Hierarchical clustering based on topological overlap dissimilarity identified multiple co-expression modules (**Figure 3b**). The brown module showed the strongest positive association with Cluster 3 (r = 0.70, *p* = 3 × 10^-72^) and was also weakly associated with deceased status (r = 0.13, *p* = 0.004; **Figure 3c**). GO enrichment analysis of the brown-module genes showed predominant enrichment in Golgi vesicle transport, vesicle tethering, vesicle targeting, post-Golgi vesicle-mediated transport, small GTPase binding, GTPase binding, and NAD(P)H-related oxidoreductase activity (**Supplementary Table 8**).

To refine the candidate gene set, we intersected genes from the brown module and the curated RCD gene set with the DEGs identified in each of the three pairwise comparisons of Cluster 3 with Clusters 1, 2, and 4. A total of 122 genes were shared across all five sets (**Figure 3d**) and were carried forward for machine-learning-based feature selection. LASSO retained 31 genes with non-zero coefficients at the selected penalty parameter (λ = 0.00379; **Figure 3e**), whereas random forest and XGBoost ranked candidate features according to MeanDecreaseGini and SHAP-based importance, respectively (**Figures 3f, g**; **Supplementary Tables 9–S11**). The intersection of the LASSO-retained genes and the top 30 features from the random forest and XGBoost models identified six consensus candidate genes: SMC4, SLC5A6, POLR3GL, MAD2L2, EXOC7, and COG1.

### Development, endotype mapping, and evaluation of a six-gene RCD.score

**Figure 4:**
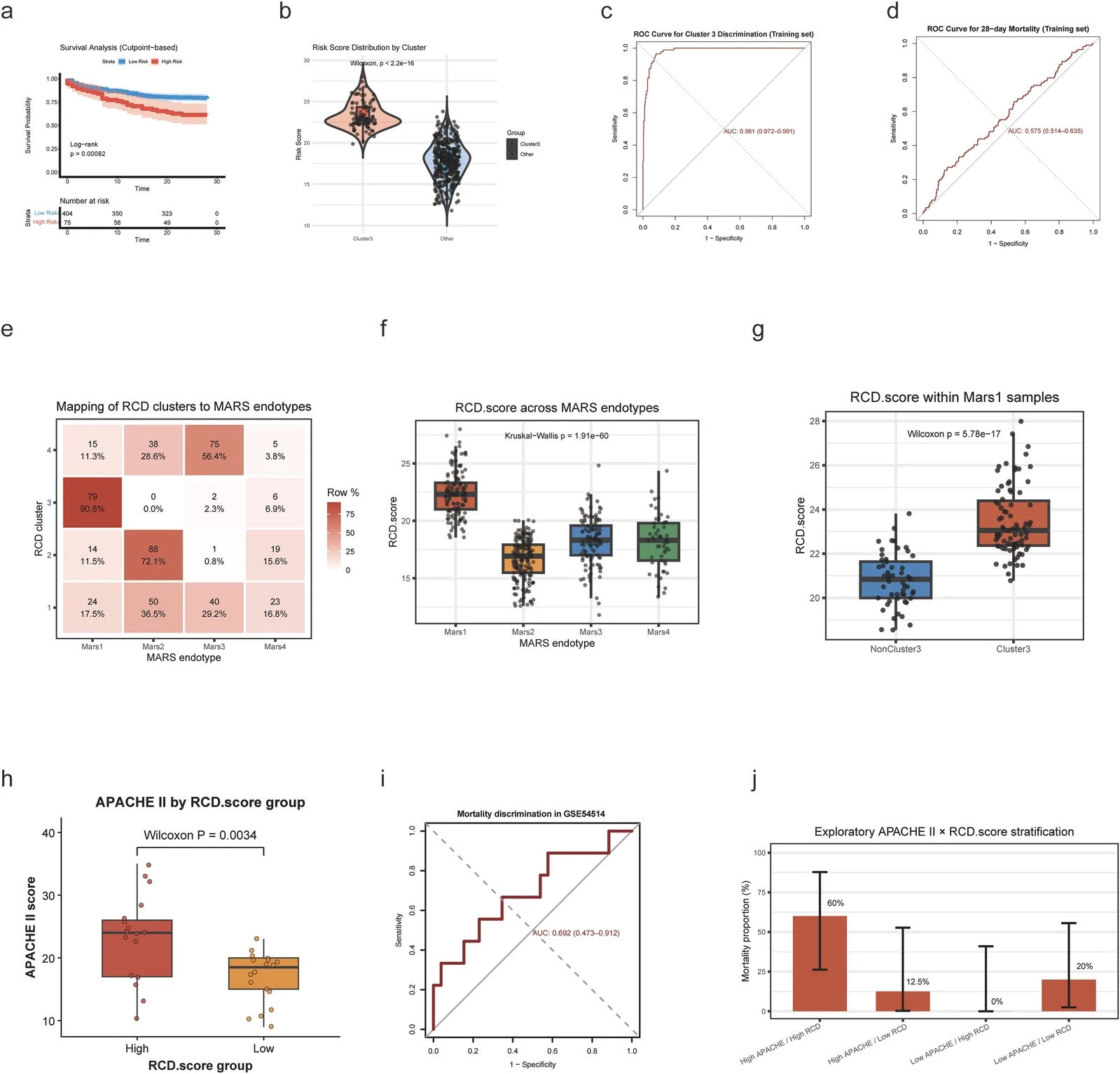
Construction, endotype mapping, and external clinical contextualization of the six-gene RCD.score. **(a)** Kaplan–Meier curves of 28-day survival in the high- and low-RCD.score groups in GSE65682. **(b)** Distribution of RCD.score in Cluster 3 and the remaining RCD-defined clusters. **(c)** Receiver operating characteristic curve for discrimination of Cluster 3 from the remaining RCD-defined clusters in GSE65682. **(d)** Receiver operating characteristic curve for discrimination of 28-day mortality by RCD.score in GSE65682. **(e)** Cross-tabulation of the RCD-defined clusters and published MARS endotypes. **(f)** Distribution of RCD.score across the four MARS endotypes. **(g)** Distribution of RCD.score among Mars1 samples assigned to Cluster 3 or to the remaining RCD-defined clusters. **(h)** APACHE II scores in the high- and low-RCD.score groups among Day 1 sepsis samples from GSE54514. **(i)** Receiver operating characteristic curve for discrimination of survivor and nonsurvivor status by the weighted RCD.score in GSE54514. **(j)** Mortality proportions across four strata jointly defined by APACHE II and weighted RCD.score in GSE54514. High APACHE II was defined as a score ≥20, and high RCD.score was defined as a value above the cohort median. RCD, regulated cell death; MARS, Molecular Diagnosis and Risk Stratification of Sepsis; APACHE II, Acute Physiology and Chronic Health Evaluation II; ROC, receiver operating characteristic; AUC, area under the curve.

A weighted RCD.score was constructed from SMC4, SLC5A6, POLR3GL, MAD2L2, EXOC7, and COG1 using their corresponding LASSO coefficients. Using an optimal cutpoint derived in the training cohort, 75 patients were classified into the high-RCD.score group and 404 into the low-RCD.score group. The high-score group had poorer 28-day survival than the low-score group (log-rank *p* = 0.00082; **Figure 4a**). RCD.score was substantially higher in Cluster 3 than in the remaining RCD-defined clusters (Wilcoxon *p* < 2.2 × 10^-16^; **Figure 4b**). The score distinguished Cluster 3 from the other clusters with an AUC of 0.981 (95% CI, 0.972–0.991; **Figure 4c**), whereas its discrimination for 28-day mortality was limited (AUC = 0.575, 95% CI, 0.514–0.635; **Figure 4d**). These findings indicate that RCD.score primarily captured the transcriptional characteristics of Cluster 3 rather than serving as a strong standalone predictor of individual mortality. We next compared the RCD-defined clusters with the published MARS endotypes in 479 patients. The two classification systems were significantly associated (χ² = 346.9, *p* = 2.84 × 10^-69^; Cramér’s V = 0.491; **Figure 4e**). Of the 87 patients in Cluster 3, 79 were classified as Mars1, accounting for 90.8% of Cluster 3; conversely, 79 of 132 Mars1 patients belonged to Cluster 3. Cluster 3 was strongly enriched within Mars1 relative to the other MARS endotypes (OR = 62.24, FDR-adjusted *p* = 3.22 × 10^-44^). RCD.score differed across the four MARS endotypes (Kruskal–Wallis *p* = 1.91 × 10^-60^; **Figure 4f**), with the highest median value observed in Mars1 (22.32), compared with Mars2 (16.93), Mars3 (18.32), and Mars4 (18.31). Within Mars1, patients assigned to Cluster 3 had higher RCD.score values than those assigned to the other RCD clusters (median, 23.05 vs 20.84; Wilcoxon *p* = 5.78 × 10^-17^; **Figure 4g**). Their 28-day mortality proportions were similar (34.2% vs 34.0%; Fisher’s exact p = 1.00). Thus, the RCD framework resolved a subgroup with stronger RCD-associated transcriptional activity within Mars1, although it did not further separate short-term mortality within that endotype.

Formal SRS classification using SepstratifieR assigned 461 patients to SRS2 and 18 to SRS3, whereas no patients were assigned to SRS1. The RCD-defined clusters were significantly associated with formal SRS classification, although the strength of association was modest (Fisher’s exact *p* = 0.0029; Cramér’s V = 0.157). RCD.score was higher in SRS3 than in SRS2 (median, 20.16 vs 18.46; Wilcoxon *p* = 0.0189), and the quantitative SRS score differed across the four RCD-defined clusters (Kruskal–Wallis *p* = 2.45 × 10^-7^). However, Cluster 3 was not significantly enriched in SRS3 after correction for multiple comparisons (OR = 1.78, 95% CI, 0.48–5.49; FDR-adjusted *p* = 0.344). Formal SRS assignments were highly consistent across alternative nearest-neighbour values, and exclusion of mutual-nearest-neighbour outliers yielded findings consistent with the primary analysis (**Supplementary Table 15**). Together, the significant quantitative associations but limited categorical overlap indicate that the RCD and SRS frameworks capture partially shared but non-identical transcriptional variation.

In sequential Cox proportional-hazards analyses including 479 patients and 114 deaths, each 1-SD increase in RCD.score was associated with a higher risk of 28-day mortality in the unadjusted model (HR 1.26, 95% CI 1.05–1.50; *p* = 0.013) and after adjustment for age and sex (HR 1.23, 95% CI 1.02–1.47; *p* = 0.029). Adding RCD.score to the age- and sex-adjusted model produced modest improvements in model fit and discrimination (likelihood-ratio *p* = 0.031; ΔAIC = −2.66; ΔC-index = 0.019). However, the association was attenuated after adjustment for MARS endotype alone (HR 1.04, 95% CI 0.78–1.39; *p* = 0.786) and after adjustment for age, sex, and MARS endotype (HR 0.99, 95% CI 0.73–1.33; *p* = 0.919). Adding RCD.score to models containing MARS did not further improve model fit or discrimination, suggesting that much of its prognostic information was shared with MARS endotyping. The proportional-hazards assumption was not violated in any model (all global *p* > 0.77; **Supplementary Figure 2**; **Supplementary Table 16**). To evaluate potential overfitting, we performed bootstrap validation of the feature-selection and score-construction procedure with 1, 000 successful patient-level resamples, repeating the feature-selection and score-construction procedure from the predefined 122-gene candidate set within each resample. For discrimination of Cluster 3 from the other clusters, the apparent AUC was 0.981 and the mean estimated optimism was 0.0069, yielding an optimism-corrected AUC of 0.974. For 28-day mortality, the apparent AUC was 0.575 and the mean estimated optimism was −0.0024, corresponding to an optimism-corrected AUC of 0.577. These results showed that discrimination of the Cluster 3 transcriptional phenotype remained high after optimism correction, while the mortality AUC was essentially unchanged, indicating minimal bootstrap-estimated optimism for both performance outcomes (**Supplementary Table 17**).

### External clinical evaluation in GSE54514

The external clinical evaluation included 35 Day 1 sepsis samples from GSE54514, including nine deaths. APACHE II scores were higher in the high-RCD.score group than in the low-RCD.score group (Wilcoxon *p* = 0.0034; **Figure 4h**). For survivor–nonsurvivor discrimination, RCD.score yielded an AUC of 0.692 (95% CI, 0.473–0.912; **Figure 4i**; **Supplementary Table 18**). Exploratory joint stratification by APACHE II and RCD.score showed the highest mortality among patients with both high APACHE II and high RCD.score. Six of 10 patients in this group died, corresponding to a mortality rate of 60.0%, compared with 12.5% in the high-APACHE II/low-RCD.score group, 0% in the low-APACHE II/high-RCD.score group, and 20.0% in the low-APACHE II/low-RCD.score group (**Figure 4j**). Compared with all remaining patients, the high-APACHE II/high-RCD.score group had a higher mortality rate (60.0% vs 12.0%; exact OR 10.00, 95% CI 1.46–91.38; Fisher’s exact *p* = 0.007). However, among patients with APACHE II ≥20, the comparison between high and low RCD.score groups did not reach statistical significance (60.0% vs 12.5%; exact OR 9.11, 95% CI 0.71–547.96; *p* = 0.066).

Adding continuous RCD.score to APACHE II did not improve overall discrimination. The AUC was 0.776 for APACHE II alone and 0.774 for APACHE II plus RCD.score (ΔAUC = −0.002; DeLong *p* = 0.950). Continuous NRI was −0.179 (bootstrap 95% CI, −0.571 to 1.071), and IDI was 0.029 (bootstrap 95% CI, −0.002 to 0.222). Thus, a global incremental benefit beyond APACHE II was not demonstrated in this small cohort. Nevertheless, the marked mortality enrichment observed in the high-APACHE II/high-RCD.score group supports further evaluation of joint clinical–molecular stratification in larger cohorts (**Supplementary Table 18**).

### High RCD.score is associated with distinct immune-signature patterns and cell-type-resolved localization to myeloid populations

**Figure 5:**
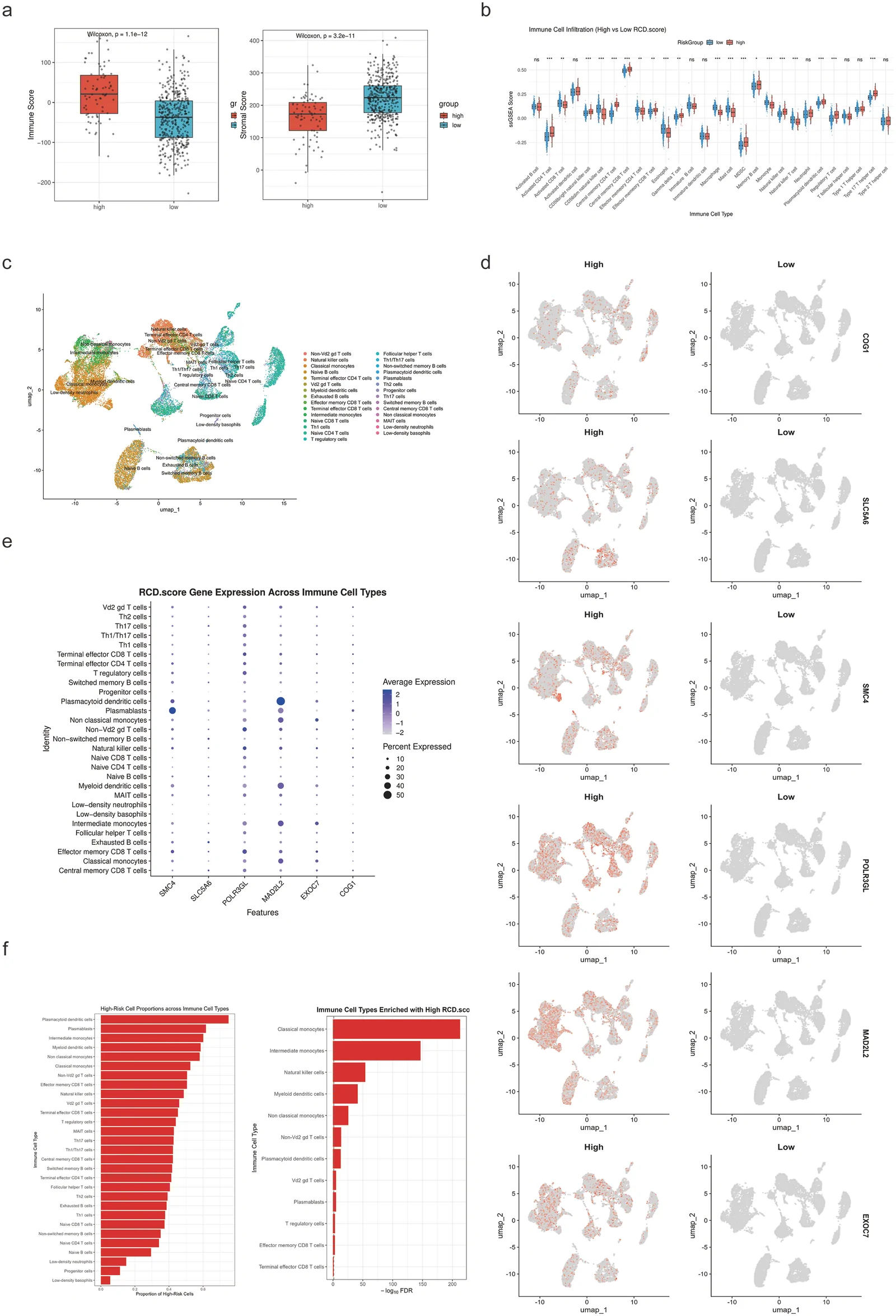
Immune-related transcriptomic patterns and single-cell mapping of the RCD.score. **(a)** ESTIMATE-derived Immune Score and Stromal Score in the high- and low-RCD.score groups in GSE65682. **(b)** ssGSEA enrichment scores for 28 immune-cell-associated signatures in the high- and low-RCD.score groups. **(c)** UMAP representation of GSE167363 showing 34 immune-cell subsets annotated using SingleR. **(d)** UMAP feature plots showing the expression of COG1, SLC5A6, SMC4, POLR3GL, MAD2L2, and EXOC7 in high- and low-RCD.score cells. **(e)** Dot plot showing expression of the six RCD.score genes across the annotated immune-cell subsets. **(f)** Distribution and enrichment of high-RCD.score cells across immune-cell subsets. Left, proportion of high-RCD.score cells within each subset. Right, enrichment relative to all other cells; dashed line, FDR = 0.05. RCD, regulated cell death; ESTIMATE, Estimation of STromal and Immune cells in MAlignant Tumor tissues using Expression data; ssGSEA, single-sample gene set enrichment analysis; UMAP, Uniform Manifold Approximation and Projection; FDR, false discovery rate.

To characterize immune-related transcriptional patterns associated with RCD.score, we compared ESTIMATE-derived scores between the high- and low-RCD.score groups. The high-RCD.score group showed a higher Immune Score and a lower Stromal Score (Wilcoxon *p* = 1.1 × 10^–12^ and *p* = 3.2 × 10^–11^, respectively; **Figure 5a**). Because these analyses were based on whole-blood transcriptomes, both metrics were interpreted as descriptive expression-derived indices rather than direct estimates of immune- or stromal-cell abundance. ssGSEA further revealed distinct immune-cell-associated signature patterns between the two groups. The high-RCD.score group showed lower scores for activated CD8 T-cell, CD56 dim natural killer-cell, monocyte, eosinophil, and macrophage signatures, whereas CD56 bright natural killer-cell, central-memory CD4 and CD8 T-cell, effector-memory CD8 T-cell, γδ T-cell, regulatory T-cell, Th17-cell, and MDSC signatures were higher (**Figure 5b**).

Single-cell analysis of GSE167363 identified 34 immune-cell subpopulations spanning lymphoid and myeloid lineages (**Figure 5c**). The six RCD.score genes were expressed across multiple immune-cell populations, with MAD2L2 showing relatively prominent expression in monocyte and dendritic-cell subsets (**Figures 5d, e**). The highest raw proportions of high-RCD.score cells were observed in plasmacytoid dendritic cells, plasmablasts, intermediate monocytes, and myeloid dendritic cells. However, formal enrichment analysis identified classical and intermediate monocytes as the most strongly enriched populations, followed by natural killer cells, myeloid dendritic cells, and non-classical monocytes (**Figure 5f**). These findings localized a substantial component of the RCD.score-associated transcriptional signal to the myeloid compartment, particularly monocyte populations.

To account for sample-level biological replication, we further evaluated the six-gene RCD.score across major immune-cell compartments in the seven samples from GSE167363. Natural killer cells showed the highest absolute RCD.score values, whereas monocytes exhibited the clearest sepsis-associated increase at the sample level (**Supplementary Figure 3a**; **Supplementary Table 19**). Decomposition of the monocyte score showed that MAD2L2 made the largest positive contribution to the sepsis-associated increase, followed by SMC4, whereas POLR3GL showed a modest negative contribution (**Supplementary Figure 3b**). Genome-wide sample-level pseudobulk differential-expression analysis, which was not restricted to the six score genes, identified 37, 305, 3, and 241 genes at FDR < 0.05 in B cells, T cells, natural killer cells, and monocytes, respectively (**Supplementary Figure 3c**; **Supplementary Table 19**). Together, these analyses supported monocytes as an important cellular compartment associated with the sepsis-related increase in RCD.score, despite natural killer cells having the highest absolute score.

### MAD2L2 is preferentially expressed in cluster 3 and is associated with innate immune and DNA replication/repair pathway signatures

**Figure 6:**
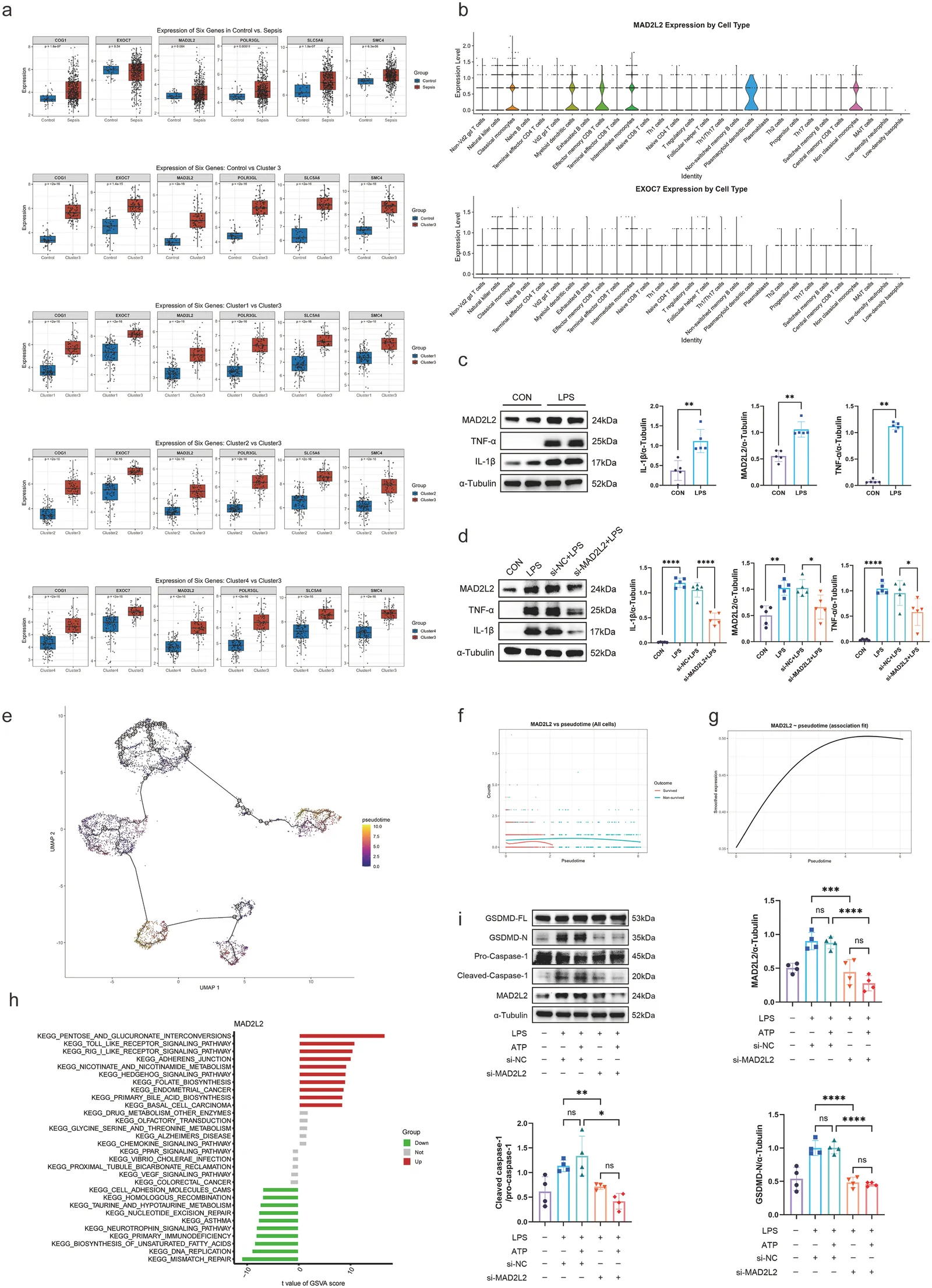
Expression and functional characterization of MAD2L2 in the Cluster 3 phenotype. **(a)** Normalized expression of the six RCD.score genes in healthy controls, the overall sepsis cohort, and the four RCD-defined subtypes. **(b)** Single-cell expression of MAD2L2 and EXOC7 across annotated immune-cell subsets, displayed as violin plots. **(c)** MAD2L2, TNF-α, and IL-1β protein expression in untreated and LPS-stimulated THP-1 cells. **(d)** MAD2L2, TNF-α, and IL-1β protein expression in THP-1 cells from the control, LPS, LPS + si-NC, and LPS + si-MAD2L2 groups. **(e)** UMAP representation of the inferred classical-monocyte trajectory colored by pseudotime. **(f)** Cell-level MAD2L2 expression across pseudotime in classical monocytes, stratified by survival status. **(g)** Fitted association curve between MAD2L2 expression and pseudotime in classical monocytes. **(h)** GSVA comparison of KEGG pathway-enrichment scores between the MAD2L2-high and MAD2L2-low groups. **(i)** GSDMD and caspase-1 protein processing in THP-1 cells from the untreated control, LPS + si-NC, LPS + ATP + si-NC, LPS + si-MAD2L2, and LPS + ATP + si-MAD2L2 groups. Representative immunoblots and densitometric quantification are shown. For panels **(c)** and **(d)**, data are presented as mean ± SD from five independent biological experiments; for panel **(i)**, data are from four independent biological experiments. Individual dots represent biological replicates, and protein abundance was normalized to α-tubulin where applicable. Statistical analyses were performed as described in the Methods. ns, not significant; *p < 0.05, **p < 0.01, ***p < 0.001, ****p < 0.0001. RCD, regulated cell death; LPS, lipopolysaccharide; ATP, adenosine triphosphate; si-NC, negative-control small interfering RNA; si-MAD2L2, MAD2L2-targeting small interfering RNA; UMAP, Uniform Manifold Approximation and Projection; GSVA, gene set variation analysis; KEGG, Kyoto Encyclopedia of Genes and Genomes; GSDMD, gasdermin D; GSDMD-FL, full-length gasdermin D; GSDMD-N, cleaved N-terminal gasdermin D; SD, standard deviation.

Bulk transcriptomic analysis revealed distinct expression patterns among the six RCD.score genes (**Figure 6a**). COG1, POLR3GL, SLC5A6, and SMC4 were expressed at higher levels in the overall sepsis cohort than in healthy controls, whereas MAD2L2 and EXOC7 did not show significant overall differences between the two groups. In contrast, all six genes were expressed at higher levels in Cluster 3 than in healthy controls and each of the other RCD-defined subtypes.

Single-cell analysis showed relatively prominent MAD2L2 expression in monocyte subsets, particularly classical monocytes, with lower expression in intermediate and non-classical monocytes, effector-memory CD8^+^ T cells, and plasmacytoid dendritic cells (**Figure 6b**). EXOC7 expression was generally low across the annotated immune-cell populations and showed no clear cell-type-specific distribution.

Pseudotime analysis mapped the inferred transcriptional trajectory of classical monocytes (**Figure 6e**). Cell-level visualization and the fitted association curve showed that MAD2L2 expression increased from early to intermediate pseudotime, subsequently approached a plateau, and decreased slightly at later pseudotime values (**Figures 6f, g**). This pattern reflects the association of MAD2L2 expression with the inferred classical-monocyte trajectory.

GSVA showed distinct pathway-enrichment patterns between the MAD2L2-high and MAD2L2-low groups (**Figure 6h**). Toll-like receptor signaling, RIG-I-like receptor signaling, Hedgehog signaling, and several metabolic pathways showed higher enrichment scores in the MAD2L2-high group. In contrast, DNA replication, mismatch repair, nucleotide excision repair, and homologous recombination showed lower enrichment scores. Thus, higher MAD2L2 expression was associated with increased innate immune-related pathway activity and reduced DNA replication/repair-related transcriptional signatures.

### MAD2L2 knockdown attenuates inflammatory and pyroptosis-associated responses in THP-1 cells

siRNA-mediated MAD2L2 knockdown efficiency in THP-1 cells was confirmed before functional analysis (**Supplementary Figure 4**). LPS stimulation increased MAD2L2, TNF-α, and IL-1β protein expression relative to untreated cells (**Figure 6c**). Silencing MAD2L2 before LPS stimulation reduced TNF-α and IL-1β expression compared with the LPS + si-NC group (**Figure 6d**), supporting the involvement of MAD2L2 in the inflammatory response of human monocyte-like cells.

We further examined GSDMD and caspase-1 processing in LPS-stimulated THP-1 cells in the presence or absence of ATP. Compared with the corresponding si-NC groups, MAD2L2 knockdown reduced GSDMD-N abundance and the cleaved caspase-1/pro-caspase-1 ratio under both LPS and LPS + ATP conditions. MAD2L2 protein expression was also reduced in the si-MAD2L2 groups, confirming effective silencing in the same experiments (**Figure 6i**). These results extend the effects of MAD2L2 knockdown from inflammatory-protein expression to pyroptosis-associated GSDMD and caspase-1 processing.

### Predicted MAD2L2-centered RNA interaction network

A predicted MAD2L2-centered RNA interaction network was constructed to explore potential post-transcriptional regulatory relationships (**Supplementary Figure 5**). The network included six candidate miRNAs—hsa-miR-194-3p, hsa-miR-296-5p, hsa-miR-890, hsa-miR-144-3p, hsa-miR-18a-3p, and hsa-miR-4290—together with their predicted interacting RNA nodes. This analysis provides a set of candidate RNA interactions for subsequent experimental investigation.

## Discussion

Sepsis is marked by pronounced clinical and molecular heterogeneity, and reliance on clinical phenotypes alone may inadequately capture the underlying mechanistic differences, thereby complicating risk stratification and the development of targeted therapies (24, 36). Recent transcriptomic studies have delineated biologically meaningful molecular subtypes that track with immune states and patient outcomes (37). We therefore developed an RCD-based molecular classification and identified Cluster 3 as a high-risk subtype with the poorest 28-day survival and a distinct immune- and cell-death-related transcriptional profile. When the input was restricted to genes associated with apoptosis, ferroptosis, necroptosis, and pyroptosis, the analysis reproduced a closely concordant four-cluster structure, and the corresponding Cluster 3 retained its adverse survival pattern. The primary classification was therefore robust to restriction of the input gene set.

Cluster 3 exhibited a mixed immune transcriptional state rather than a uniform pattern of immune activation or suppression. Central-memory CD4^+^ and CD8^+^ T-cell and Th17-cell signatures were consistently higher in Cluster 3 than in the other subtypes. Higher scores were also observed for several activation- or regulation-associated signatures, including activated CD4^+^ T cells, CD56 bright natural killer cells, effector-memory CD8^+^ T cells, regulatory T cells, and MDSCs, although the direction and magnitude of these differences varied across pairwise comparisons. In contrast, CD56 dim natural killer-cell, macrophage, and mast-cell signatures were lower in Cluster 3. Such a mixed pattern accords with evidence that inflammatory and immunosuppressive programs can coexist during sepsis (38, 39). ESTIMATE-derived Immune and Stromal Scores were treated as supporting expression-based measures. Because the analysis was conducted in whole blood, the lower Stromal Score was not interpreted as evidence of stromal depletion, structural damage, or reduced tissue integrity.

Single-cell analysis localized a substantial component of the RCD.score-associated signal to the myeloid compartment, particularly classical and intermediate monocytes, with additional enrichment in myeloid dendritic cells. This distribution is consistent with the established importance of myeloid dysfunction in sepsis. Dendritic-cell loss and functional impairment have been associated with secondary infection and adverse outcomes (40), whereas reduced monocytic HLA-DR expression, a widely used marker of monocyte deactivation, has been linked to greater disease severity and poorer prognosis (41, 42). These established roles provide biological context for the observed cellular distribution of RCD.score and highlight monocyte and dendritic-cell compartments as relevant cellular contexts for the high-risk phenotype.

The sample-level analysis further clarified this cell-type pattern. Although natural killer cells had the highest absolute RCD.score values, monocytes showed the clearest increase in baseline sepsis samples relative to healthy controls. Among the six score genes, MAD2L2 made the largest positive contribution to this monocyte-associated increase, followed by SMC4, which supported the use of a monocytic cell model for subsequent experiments. Cell-type-specific pseudobulk analysis also showed that the transcriptional response to sepsis varied across immune compartments, with more differentially expressed genes detected in T cells and monocytes than in natural killer cells. These findings further identify monocytes as an important cellular setting for understanding the RCD.score phenotype and evaluating MAD2L2.

The six-gene RCD.score closely reflected the transcriptional pattern of Cluster 3 and also retained prognostic relevance in sequential Cox analyses. Each 1-SD increase in RCD.score was associated with higher 28-day mortality in the unadjusted and age- and sex-adjusted models, and its addition to the age- and sex-adjusted model modestly improved model fit and discrimination. The association was attenuated after MARS endotype was included, which, together with the marked overlap between Cluster 3 and Mars1, suggests that RCD.score and MARS capture partly shared host-response information. Their classifications were not identical, however, and the relationship between the RCD framework and formal SRS classification was weaker, with no significant enrichment of Cluster 3 in SRS3 after multiple-testing correction. Bootstrap validation showed minimal optimism for discrimination of Cluster 3, supporting the stability of RCD.score at the feature-selection and score-construction stage. Because the bootstrap procedure began from the predefined candidate-gene set, these results specifically support the internal robustness of the derived score rather than independent replication of the upstream subtype-discovery process. Thus, although the RCD and MARS classifications partly overlapped, the RCD analysis provided a cell-death-focused view of the high-risk subtype and linked it to cell-type-specific patterns and the experimental findings on MAD2L2.

In GSE54514, patients with higher RCD.score values had higher APACHE II scores, and RCD.score showed moderate discrimination between survivors and nonsurvivors. Although adding continuous RCD.score to APACHE II did not improve overall AUC, NRI, or IDI, exploratory joint stratification identified a risk-enriched subgroup: patients with both APACHE II ≥20 and high RCD.score had 60.0% mortality, compared with 12.0% among all remaining patients. This pattern suggests that the molecular phenotype captured by RCD.score may provide complementary molecular context when considered alongside clinical severity. RCD.score should therefore be interpreted as a molecular descriptor of the Cluster 3 phenotype and a candidate component of combined clinical–molecular stratification rather than as a replacement for established clinical severity scores. This distinction is biologically plausible because short-term outcome in sepsis reflects not only the circulating host response but also age, comorbidities, infection source, organ dysfunction, and subsequent treatment. Previous prospective studies have shown that blood transcriptomic programs capture clinically meaningful differences in sepsis host response and outcome (43), and recent harmonization work suggests that apparently distinct transcriptomic classifications share reproducible biological structure across cohorts (44). Our findings place the RCD-based classification within this broader framework while adding a cell-death-oriented view of the high-risk phenotype.

Among the six genes included in the RCD.score, MAD2L2 was notable because its expression was selectively elevated in Cluster 3 rather than broadly increased across the sepsis cohort. Its prominent expression in monocyte subsets and its contribution to the sepsis-associated increase in monocyte RCD.score provided the rationale for further evaluation in a monocytic cell model. In THP-1 cells, LPS stimulation increased MAD2L2, TNF-α, and IL-1β protein expression, whereas MAD2L2 knockdown reduced these inflammatory responses. In the extended experiments, MAD2L2 silencing also decreased GSDMD-N abundance and the cleaved caspase-1/pro-caspase-1 ratio under both LPS and LPS + ATP conditions. These findings indicate that the effect of MAD2L2 knockdown extends beyond inflammatory-protein expression to pyroptosis-associated GSDMD and caspase-1 processing.

MAD2L2 is an established component of the 53BP1–RIF1–shieldin axis, where it limits DNA-end resection and promotes non-homologous end joining. It also participates in polymerase-zeta-mediated translesion synthesis, replication-fork protection, and replication restart, linking MAD2L2 to the maintenance of genome stability (45–47). In classical monocytes, MAD2L2 expression increased from early to intermediate pseudotime and then approached a plateau. The MAD2L2-high group also showed higher enrichment scores for Toll-like receptor and RIG-I-like receptor signaling, together with lower scores for DNA replication and several DNA-repair pathways, including homologous recombination. DNA damage can engage innate immune signaling through cytosolic DNA-sensing pathways such as cGAS–STING (48, 49), which has also been implicated in inflammatory organ injury during sepsis (50, 51). Taken together, the transcriptomic and experimental findings support the hypothesis that MAD2L2-associated changes in genome-maintenance programs may be linked to innate immune and pyroptosis-related responses in monocytes.

### Limitations

Several limitations should be considered. First, the study relied primarily on publicly available whole-blood transcriptomic datasets. Differences in patient populations, sampling procedures, and expression platforms may have introduced residual heterogeneity despite standardized preprocessing and external evaluation. In addition, because ESTIMATE was originally developed for tissue transcriptomes, the Stromal Score in whole blood was treated as a descriptive expression-derived measure rather than a direct indicator of stromal-cell abundance or tissue structural integrity.

Second, functional experiments were performed in THP-1 monocyte-like cells. MAD2L2 knockdown reduced LPS-induced TNF-α and IL-1β expression and was also accompanied by lower GSDMD-N abundance and cleaved caspase-1/pro-caspase-1 ratios under both LPS and LPS + ATP conditions, extending the experimental validation to pyroptosis-associated protein processing. Because these experiments were conducted in a single monocytic cell line, the functional findings should be considered hypothesis-generating. Further validation in primary human monocytes or macrophages and *in vivo* models will help determine whether the observed effects are reproduced within the broader septic immune environment and how they relate to DNA-damage-associated signaling.

Third, the external clinical evaluation included only 35 patients and nine deaths, and SOFA was unavailable in the deposited clinical annotations. These constraints limited the precision of the incremental analyses and prevented comparison with multiple established severity scores. Although joint stratification by APACHE II and RCD.score identified a subgroup with markedly higher observed mortality, this risk-enrichment finding requires confirmation in larger prospective cohorts with broader clinical characterization and sufficient outcome events.

Finally, the single-cell analysis provided descriptive cell-type mapping and sample-level contextualization rather than independent validation of the bulk-derived signature. Given the limited number of healthy control samples and potential cross-platform differences, confirmation in larger independent single-cell cohorts, ideally with matched bulk and single-cell profiles, is warranted.

## Conclusions

RCD-based molecular subtyping identified a high-risk sepsis subtype characterized by a distinct immune transcriptional profile and prominent localization of the RCD.score-associated signal to monocyte populations. The six-gene RCD.score captured the molecular features of this subtype and enabled its cell-type-resolved characterization. MAD2L2 was preferentially expressed in Cluster 3 and monocyte subsets. In THP-1 cells, MAD2L2 knockdown reduced LPS-induced TNF-α and IL-1β expression and decreased GSDMD-N abundance and caspase-1 processing under both LPS and LPS + ATP conditions. These findings support MAD2L2 as a candidate involved in monocyte inflammatory and pyroptosis-associated responses in sepsis.

## Data Availability

The datasets analyzed in this study are publicly available in the Gene Expression Omnibus (GEO) database under accession numbers GSE65682, GSE54514, and GSE167363. All other data supporting the findings of this study are available from the corresponding author upon reasonable request.

## Glossary

Abbreviations: Description
RCD: Regulated cell-death
ssGSEA: single-sample gene set enrichment analysis
WGCNA: Weighted gene co-expression network analysis
LASSO: Least absolute shrinkage and selection operator
XGBoost: eXtreme Gradient Boosting
APACHE II: Acute Physiology and Chronic Health Evaluation II
SMC4: Structural Maintenance of Chromosomes 4
SLC5A6: Solute Carrier Family 5 Member 6
POLR3GL: RNA Polymerase III Subunit G
MAD2L2: Mitotic Arrest Deficient 2 Like 2
EXOC7: Exocyst Complex Component 7
COG1: Component of Oligomeric Golgi Complex 1
SOFA: Sequential Organ Failure Assessment
MARS: Molecular Diagnosis and Risk Stratification of Sepsis
SRS: Sepsis Response Signature
SRSq: Quantitative Sepsis Response Signature score
FDR: False discovery rate
DEGs: Differentially expressed genes
GO: Gene Ontology
KEGG: Kyoto Encyclopedia of Genes and Genomes
ESTIMATE: Estimation of STromal and Immune cells in MAlignant Tumour tissues using Expression data
GSVA: Gene set variation analysis
TOM: Topological overlap matrices
ME: Module eigengene
MM: Module membership
GS: Gene significance
PCA: Principal component analysis
UMAP: Uniform Manifold Approximation and Projection
ROC: Receiver operating characteristic
pDCs: plasmacytoid dendritic cells
Tregs: regulatory T cells
MDSCs: Myeloid-derived suppressor cells
AIC: Akaike information criterion
NRI: Net reclassification improvement
IDI: Integrated discrimination improvement

## References

[1] Rudd KE Johnson SC Agesa KM Shackelford KA Tsoi D Kievlan DR . Global, regional, and national sepsis incidence and mortality, 1990-2017: analysis for the Global Burden of Disease Study. Lancet. (2020) 395:200–11. doi: 10.1016/s0140-6736(19)32989-7 31954465 PMC6970225

[2] Evans L Rhodes A Alhazzani W Antonelli M Coopersmith CM French C . Surviving sepsis campaign: international guidelines for management of sepsis and septic shock 2021. Intensive Care Med. (2021) 47:1181–247. doi: 10.1097/ccm.0000000000005337 34599691 PMC8486643

[3] Vincent JL Moreno R Takala J Willatts S De Mendonça A Bruining H . The SOFA (Sepsis-related Organ Failure Assessment) score to describe organ dysfunction/failure. Intensive Care Med. (1996) 22:707–10. doi: 10.1007/bf01709751 8844239

[4] Seymour CW Kennedy JN Wang S Chang CH Elliott CF Xu Z . Derivation, validation, and potential treatment implications of novel clinical phenotypes for sepsis. Jama. (2019) 321:2003–17. doi: 10.1001/jama.2019.5791 31104070 PMC6537818

[5] Galluzzi L Vitale I Aaronson SA Abrams JM Adam D Agostinis P . Molecular mechanisms of cell death: recommendations of the Nomenclature Committee on Cell Death 2018. Cell Death Differ. (2018) 25:486–541. doi: 10.1038/s41418-017-0012-4 29362479 PMC5864239

[6] Hotchkiss RS Monneret G Payen D . Immunosuppression in sepsis: a novel understanding of the disorder and a new therapeutic approach. Lancet Infect Dis. (2013) 13:260–8. doi: 10.1016/s1473-3099(13)70001-x 23427891 PMC3798159

[7] Remick DG . Pathophysiology of sepsis. Am J Pathol. (2007) 170:1435–44. doi: 10.2353/ajpath.2007.060872 17456750 PMC1854939

[8] Vasudevan SO Behl B Rathinam VA . Pyroptosis-induced inflammation and tissue damage. Semin Immunol. (2023) 69:101781. doi: 10.1016/j.smim.2023.101781 37352727 PMC10598759

[9] Nie J Zhou L Tian W Liu X Yang L Yang X . Deep insight into cytokine storm: from pathogenesis to treatment. Signal Transduction Targeted Ther. (2025) 10:112. doi: 10.1038/s41392-025-02178-y 40234407 PMC12000524

[10] Xiao Z Zhang J Qiu Z Liu H Ding H Li H . Ferroptosis and inflammation are modulated by the NFIL3-ACSL4 axis in sepsis associated-acute kidney injury. Cell Death Discov. (2024) 10:349. doi: 10.1038/s41420-024-02113-0 39097582 PMC11297963

[11] Yang H Cui Y Peng W Zhu F Ma S Rao M . Identification of molecular subtypes and a novel prognostic model of sepsis based on ferroptosis-associated gene signature. Biomolecules. (2022) 12:1479. doi: 10.3390/biom12101479 36291692 PMC9599462

[12] Peng S Meng N Xie X Zhu B Wang B . Identification of subtypes and diagnostic markers related to necroptosis in sepsis. Appl Biochem Biotechnol. (2025) 197:3689–705. doi: 10.1007/s12010-025-05201-8 40009340 PMC12170754

[13] Langfelder P Horvath S . WGCNA: an R package for weighted correlation network analysis. BMC Bioinf. (2008) 9:559. doi: 10.1186/1471-2105-9-559 19114008 PMC2631488

[14] Sweeney TE Shidham A Wong HR Khatri P . A comprehensive time-course-based multicohort analysis of sepsis and sterile inflammation reveals a robust diagnostic gene set. Sci Transl Med. (2015) 7:287ra71. doi: 10.1126/scitranslmed.aaa5993 25972003 PMC4734362

[15] Shen Z Zhang S Jiao Y Shi Y Zhang H Wang F . LASSO model better predicted the prognosis of DLBCL than random forest model: A retrospective multicenter analysis of HHLWG. J Oncol. (2022) 2022:1618272. doi: 10.1155/2022/1618272 36157230 PMC9507678

[16] Xiao B Yang M Meng Y Wang W Chen Y Yu C . Construction of a prognostic prediction model for colorectal cancer based on 5-year clinical follow-up data. Sci Rep. (2025) 15:2701. doi: 10.1038/s41598-025-86872-5 39838027 PMC11750956

[17] Yin JM Li Y Xue JT Zong GW Fang ZZ Zou L . Explainable machine learning-based prediction model for diabetic nephropathy. J Diabetes Res. (2024) 2024:8857453. doi: 10.1155/2024/8857453 38282659 PMC10821806

[18] Barrett T Wilhite SE Ledoux P Evangelista C Kim IF Tomashevsky M . NCBI GEO: archive for functional genomics data sets—update. Nucleic Acids Res. (2012) 41:D991–5. doi: 10.1093/nar/gks1193 23193258 PMC3531084

[19] Davis S Meltzer PS . GEOquery: a bridge between the gene expression omnibus (GEO) and bioconductor. Bioinf (Oxford England). (2007) 23:1846–7. doi: 10.1093/bioinformatics/btm254 17496320

[20] Ritchie ME Phipson B Wu D Hu Y Law CW Shi W . Limma powers differential expression analyses for RNA-sequencing and microarray studies. Nucleic Acids Res. (2015) 43:e47. doi: 10.1093/nar/gkv007 25605792 PMC4402510

[21] Zhang W Zhu Y Liu H Zhang Y Liu H Adegboro AA . Pan-cancer evaluation of regulated cell death to predict overall survival and immune checkpoint inhibitor response. NPJ Precis Oncol. (2024) 8:77. doi: 10.1038/s41698-024-00570-5 38538696 PMC10973470

[22] Wilkerson MD Hayes DN . ConsensusClusterPlus: a class discovery tool with confidence assessments and item tracking. Bioinf (Oxford England). (2010) 26:1572–3. doi: 10.1093/bioinformatics/btq170 20427518 PMC2881355

[23] Scicluna BP Van Vught LA Zwinderman AH Wiewel MA Davenport EE Burnham KL . Classification of patients with sepsis according to blood genomic endotype: a prospective cohort study. Lancet Respir Med. (2017) 5:816–26. doi: 10.1016/s2213-2600(17)30294-1 28864056

[24] Davenport EE Burnham KL Radhakrishnan J Humburg P Hutton P Mills TC . Genomic landscape of the individual host response and outcomes in sepsis: a prospective cohort study. Lancet Respir Med. (2016) 4:259–71. doi: 10.1016/s2213-2600(16)00046-1 26917434 PMC4820667

[25] Kaplan EL Meier P . Nonparametric estimation from incomplete observations. J Am Stat Assoc. (1958) 53:457–81. doi: 10.1080/01621459.1958.10501452 37339054

[26] Yu G Wang L-G Han Y He Q-Y . ClusterProfiler: an R package for comparing biological themes among gene clusters. Omics: A J Integr Biol. (2012) 16:284–7. doi: 10.1089/omi.2011.0118 22455463 PMC3339379

[27] Yoshihara K Shahmoradgoli M Martínez E Vegesna R Kim H Torres-Garcia W . Inferring tumour purity and stromal and immune cell admixture from expression data. Nat Commun. (2013) 4:2612. doi: 10.1038/ncomms3612 24113773 PMC3826632

[28] Hänzelmann S Castelo R Guinney J . GSVA: gene set variation analysis for microarray and RNA-seq data. BMC Bioinf. (2013) 14:7. 10.1186/1471-2105-14-7 PMC3618321 23323831

[29] Friedman J Hastie T Tibshirani R . Regularization paths for generalized linear models via coordinate descent. J Stat Software. (2010) 33:1–22. doi: 10.18637/jss.v033.i01 PMC2929880 20808728

[30] Liaw A Wiener M . Classification and regression by randomForest. R News. (2002) 2:18–22.

[31] Chen T Guestrin C . (2016). “ XGBoost: a scalable tree boosting system”, in: Proceedings of the 22nd ACM SIGKDD International Conference on Knowledge Discovery and Data Mining (San Francisco, California, USA: Association for Computing Machinery), 785–94.

[32] Qiu X Li J Bonenfant J Jaroszewski L Mittal A Klein W . Dynamic changes in human single-cell transcriptional signatures during fatal sepsis. J Leukocyte Biol. (2021) 110:1253–68. doi: 10.1002/jlb.5ma0721-825r 34558746 PMC8629881

[33] Hao Y Hao S Andersen-Nissen E Mauck WM Zheng S Butler A . Integrated analysis of multimodal single-cell data. Cell. (2021) 184:3573–87. doi: 10.1016/j.cell.2021.04.048 34062119 PMC8238499

[34] Aran D Looney AP Liu L Wu E Fong V Hsu A . Reference-based analysis of lung single-cell sequencing reveals a transitional profibrotic macrophage. Nat Immunol. (2019) 20:163–74. doi: 10.1038/s41590-018-0276-y 30643263 PMC6340744

[35] Monaco G Lee B Xu W Mustafah S Hwang YY Carré C . RNA-Seq signatures normalized by mRNA abundance allow absolute deconvolution of human immune cell types. Cell Rep. (2019) 26:1627–40. doi: 10.1016/j.celrep.2019.01.041 30726743 PMC6367568

[36] Sinha P Kerchberger VE Willmore A Chambers J Zhuo H Abbott J . Identifying molecular phenotypes in sepsis: an analysis of two prospective observational cohorts and secondary analysis of two randomised controlled trials. Lancet Respir Med. (2023) 11:965–74. doi: 10.1016/s2213-2600(23)00237-0 37633303 PMC10841178

[37] Cano-Gamez K Burnham KL Goh C Allcock A Malick ZH Overend L . An immune dysfunction score for stratification of patients with acute infection based on whole-blood gene expression. Sci Transl Med. (2022) 14:eabq4433. doi: 10.1126/scitranslmed.abq4433 36322631 PMC7613832

[38] Hotchkiss RS Moldawer LL Opal SM Reinhart K Turnbull IR Vincent JL . Sepsis and septic shock. Nat Rev Dis Primers. (2016) 2:16045. doi: 10.1038/nrdp.2016.45 28117397 PMC5538252

[39] Wiersinga WJ van der Poll T . Immunopathophysiology of human sepsis. EBioMedicine. (2022) 86:104363. doi: 10.1016/j.ebiom.2022.104363 36470832 PMC9783164

[40] Zheng LY Duan Y He PY Wu MY Wei ST Du XH . Dysregulated dendritic cells in sepsis: functional impairment and regulated cell death. Cell Mol Biol Lett. (2024) 29:81. doi: 10.1186/s11658-024-00602-9 38816685 PMC11140885

[41] Santacroce E D’Angerio M Ciobanu AL Masini L Lo Tartaro D Coloretti I . Advances and challenges in sepsis management: modern tools and future directions. Cells. (2024) 13:439. doi: 10.3390/cells13050439 38474403 PMC10931424

[42] Cui J Cai W Zhang L Wu Y Huang Y Zhao W . Decreased monocytic HLA-DR in patients with sepsis: prediction of diagnosis, severity and prognosis. Clin Biochem. (2025) 135:110851. doi: 10.1016/j.clinbiochem.2024.110851 39550023

[43] Galtung N Diehl-Wiesenecker E Lehmann D Markmann N Bergström WH Wacker J . Prospective validation of a transcriptomic severity classifier among patients with suspected acute infection and sepsis in the emergency department. Eur J Emergency Medicine: Off J Eur Soc For Emergency Med. (2022) 29:357–65. doi: 10.1097/mej.0000000000000931 35467566 PMC9432813

[44] Scicluna BP Cano-Gamez K Burnham KL Davenport EE Moore AR Khan S . A consensus blood transcriptomic framework for sepsis. Nat Med. (2025) 31:4119–30. doi: 10.1038/s41591-025-03964-5 41028542 PMC12705454

[45] Noordermeer SM Adam S Setiaputra D Barazas M Pettitt SJ Ling AK . The shieldin complex mediates 53BP1-dependent DNA repair. Nature. (2018) 560:117–21. doi: 10.1038/s41586-018-0340-7 30022168 PMC6141009

[46] Paniagua I Tayeh Z Falcone M Hernández Pérez S Cerutti A Jacobs JJL . MAD2L2 promotes replication fork protection and recovery in a shieldin-independent and REV3L-dependent manner. Nat Commun. (2022) 13:5167. doi: 10.1038/s41467-022-32861-5 36075897 PMC9458726

[47] Paniagua I Jacobs JJL . Freedom to err: the expanding cellular functions of translesion DNA polymerases. Mol Cell. (2023) 83:3608–21. doi: 10.1016/j.molcel.2023.07.008 37625405

[48] Maggs LR McVey M . REV7: a small but mighty regulator of genome maintenance and cancer development. Front Oncol. (2025) 14:1516165. doi: 10.3389/fonc.2024.1516165 39839778 PMC11747621

[49] He M Jiang H Li S Xue M Wang H Zheng C . The crosstalk between DNA-damage responses and innate immunity. Int Immunopharmacol. (2024) 140:112768. doi: 10.1016/j.intimp.2024.112768 39088918

[50] Han Y Qiu L Wu H Song Z Ke P Wu X . Focus on the cGAS-STING signaling pathway in sepsis and its inflammatory regulatory effects. J Inflammation Res. (2024) 17:3629–39. doi: 10.2147/jir.s465978 38855170 PMC11162626

[51] Tong J Song J Zhang W Zhai J Guan Q Wang H . When DNA-damage responses meet innate and adaptive immunity. Cell Mol Life Sciences: CMLS. (2024) 81:185. doi: 10.1007/s00018-024-05214-2 38630271 PMC11023972

