# Supplementary material for: A high-risk sepsis subtype identified by regulated cell death signatures and MAD2L2 validation
Source: Front Immunol. 2026 Aug 27;17:1846661. doi: 10.3389/fimmu.2026.1846661 (PMC13557957; doi:10.3389/fimmu.2026.1846661)
Supplement: Supplementary file 2 [file Table2.docx]

**Supplementary Methods**

**Survival, differential-expression, and functional-enrichment analyses**

Survival duration and vital status were obtained from the clinical annotations of GSE65682, with status coded as 0 for alive and 1 for deceased. Time-to-event objects were constructed using the Surv function in the survival package. Kaplan–Meier curves were fitted using survfit and visualized with 95% confidence intervals and numbers at risk. Differences between survival curves were evaluated using log-rank tests. For the training-cohort comparison of high- and low-RCD.score groups, the cutoff was determined using the maximally selected rank-statistic procedure implemented in surv cutpoint; this cutoff was used only within GSE65682.

Differential-expression analyses were performed using limma. Design matrices without an intercept were constructed according to the relevant sample groups, and prespecified pairwise contrasts were fitted using lmFit and contrasts.fit, followed by empirical Bayes moderation with eBayes. Unless otherwise specified, differentially expressed genes were defined by an absolute log₂ fold change greater than 1 and a Benjamini–Hochberg-adjusted false-discovery rate below 0.05. Positive log₂ fold-change values were interpreted according to the direction of the corresponding contrast.

For heat-map visualization, expression values were standardized by gene. Samples were ordered according to molecular-subtype assignment, and sample clustering was disabled when a cluster-ordered display was required. Gene Ontology over-representation analyses were performed separately for Biological Process, Cellular Component, and Molecular Function categories using clusterProfiler and org.Hs.eg.db. KEGG enrichment analyses were performed on the corresponding differentially expressed gene lists. Enrichment results were ranked and summarized according to Benjamini–Hochberg-adjusted p values.

**Primary RCD gene-compendium curation**

The primary RCD compendium was adopted from Zhang et al., who assembled modality-specific signatures for 18 forms of regulated cell death from public databases and published literature and merged multiple source lists within each modality. The present study did not perform de novo expansion of the compendium or select genes according to their associations with sepsis, molecular-subtype assignment, or survival. Genes assigned to at least one RCD modality in the published compendium were eligible for inclusion. Duplicate gene symbols were counted once in the clustering matrix, while all original modality assignments were preserved in Supplementary Table S1. Genes without a corresponding expression measurement in the processed GSE65682 matrix were excluded only at the platform-matching stage. Consequently, the primary compendium contained 7,460 unique genes, of which 4,929 were included in the primary consensus-clustering analysis.

**Additional WGCNA parameters**

Before network construction, the processed gene-level expression matrix described in the main Methods was used. Genes with a standard deviation of 0.7 or less across samples were excluded, leaving 2,463 genes for weighted gene co-expression network analysis.

Gene and sample quality was evaluated using goodSamplesGenes. Samples were hierarchically clustered using average linkage, and potential outliers were assessed using a static cut height of 20,000. No samples were removed after this quality-control step.

Candidate soft-thresholding powers from 1 to 20 were assessed using pickSoftThreshold. A soft-thresholding power of 10 was selected based on the balance between approximate scale-free topology and mean connectivity. An unsigned adjacency matrix was constructed and transformed into a topological overlap matrix. Modules were identified by dynamic tree cutting using a minimum module size of 60, deepSplit = 2, and pamRespectsDendro = FALSE. Modules with similar eigengene profiles were merged using a cut height of 0.25.

Module eigengenes were correlated with molecular-subtype indicators, survival time, and vital status. Vital status was coded as 0 for alive and 1 for deceased. Module membership was calculated as the correlation between individual gene expression and the corresponding module eigengene, whereas gene significance represented the correlation between gene expression and the trait of interest.

**Restricted RCD gene-set sensitivity analysis**

To evaluate whether the primary molecular classification depended on broad or peripheral RCD annotations, a restricted gene set was constructed using four widely studied RCD modalities: apoptosis, ferroptosis, necroptosis, and pyroptosis. The source compendium contained 907 apoptosis-, 788 ferroptosis-, 315 necroptosis-, and 624 pyroptosis-associated gene–modality records, yielding 2,634 records in total.

Because some genes were assigned to more than one RCD modality, duplicated gene symbols were counted once for clustering while their original modality annotations were retained in the supplementary source table. After deduplication, 2,255 unique genes remained, of which 1,616 were represented in the processed GSE65682 expression matrix.

Expression values were standardized by gene, and consensus clustering was repeated in the same 479 patients. To allow direct assessment of classification robustness, the number of clusters was prespecified as four rather than reselected. All other clustering parameters were identical to those used in the primary analysis, including 100 resampling iterations, pItem = 0.8, pFeature = 1.0, k-means clustering, and Euclidean distance.

Restricted clusters were matched to the corresponding primary clusters according to maximal sample overlap. Overall concordance was quantified using the adjusted Rand index and normalized mutual information. Additional measures included the proportion of patients assigned to corresponding clusters, the retention rate of primary Cluster 3, the purity of restricted Cluster 3, and the Jaccard index. The Cluster 3 retention rate was defined as the proportion of patients in primary Cluster 3 who remained in the corresponding restricted Cluster 3. Purity was defined as the proportion of restricted Cluster 3 patients who originated from primary Cluster 3. The Jaccard index was calculated as the number of patients shared by primary and restricted Cluster 3 divided by the number assigned to either cluster.

Twenty-eight-day survival was compared across the four restricted clusters using Kaplan–Meier curves and an overall log-rank test. The restricted cluster corresponding to primary Cluster 3 was additionally compared with the remaining restricted clusters using Kaplan–Meier analysis, the log-rank test, and a univariable Cox proportional-hazards model. The proportional-hazards assumption for the Cox model was evaluated using Schoenfeld residuals.

**Comparison with MARS and formal SRS transcriptomic endotypes**

Published MARS annotations in GSE65682 were matched to the RCD-defined clusters at the sample level. Associations between the two classification systems were evaluated using contingency tables and the χ² test or Fisher’s exact test, as appropriate. The strength of association was quantified using Cramér’s V. Enrichment of Cluster 3 within individual MARS endotypes was evaluated using Fisher’s exact tests, followed by Benjamini–Hochberg correction.

RCD.score distributions across the four MARS endotypes were compared using the Kruskal–Wallis test. Because Cluster 3 showed substantial overlap with Mars1, an additional within-Mars1 analysis compared Cluster 3 with the remaining RCD clusters. RCD.score distributions were compared using the Wilcoxon rank-sum test, and 28-day mortality proportions were compared using Fisher’s exact test.

Formal Sepsis Response Signature classification was performed using SepstratifieR and the published Davenport seven-gene signature. Historical gene symbols were harmonized before classification. The primary nearest-neighbour parameter was set to k = 101, corresponding to approximately 21% of the 479-patient cohort. For each patient, SepstratifieR generated a formal SRS assignment, a quantitative SRS score, and a mutual-nearest-neighbour outlier indicator.

Associations between formal SRS classification and RCD clusters were assessed using contingency tables, Fisher’s exact tests, and Cramér’s V. RCD.score was compared between SRS groups using the Wilcoxon rank-sum test, whereas the quantitative SRS score was compared across the four RCD clusters using the Kruskal–Wallis test. Cluster 3 enrichment within SRS groups was evaluated using Fisher’s exact tests with Benjamini–Hochberg correction.

Sensitivity analyses were performed using alternative nearest-neighbour values of k = 72, 96, 120, and 144, corresponding to approximately 15%, 20%, 25%, and 30% of the cohort. Agreement with the primary k = 101 classification was calculated for each alternative value. The principal comparisons were also repeated after excluding patients identified as mutual-nearest-neighbour outliers.

**Sequential Cox modeling and bootstrap validation of score construction**

The association between RCD.score and 28-day mortality was evaluated in 479 patients, including 114 deaths. Eleven deaths recorded on day 0 were retained by assigning a follow-up time of 0.5 days. RCD.score was standardized before Cox analysis so that hazard ratios represented the association per 1-SD increase.

Four sequential Cox proportional-hazards models were examined: an unadjusted model containing RCD.score; a model adjusted for age and sex; a model adjusted for MARS endotype; and a model adjusted for age, sex, and MARS endotype. MARS endotype was entered as a categorical variable.

For each adjustment set, the model containing RCD.score was compared with the corresponding baseline model without RCD.score. Incremental model fit was assessed using likelihood-ratio tests and changes in Akaike information criterion. Discrimination was assessed using Harrell’s C-index and the corresponding change in C-index after addition of RCD.score. Proportional-hazards assumptions were assessed using Schoenfeld residuals and the global proportional-hazards test.

Potential optimism was assessed through patient-level bootstrap resampling. In each replicate, patients were sampled with replacement, and the feature-selection and score-construction steps for discrimination of Cluster 3 were repeated starting from the predefined 122-gene candidate set. The resulting bootstrap-derived score was evaluated in both the bootstrap sample and the original cohort for discrimination of Cluster 3 and 28-day mortality. Optimism was defined separately for each performance outcome as the difference between the corresponding bootstrap-sample and original-cohort AUCs.

Mean optimism was calculated across 1,000 successful bootstrap replicates, and the optimism-corrected AUC for each outcome was obtained by subtracting mean optimism from the corresponding apparent AUC.

**External clinical contextualization in GSE54514**

The external analysis was restricted to Day 1 sepsis samples from GSE54514 to avoid repeated observations arising from serial sampling. Patients were required to have complete expression measurements for the six RCD.score genes, APACHE II scores, and survivor/nonsurvivor status. The final analysis comprised 35 patients, including nine nonsurvivors.

The fixed six-gene composition and coefficients derived entirely from GSE65682 were applied to the normalized GSE54514 expression data. The RCD.score was not refitted in the external cohort, and no additional feature selection or coefficient re-estimation was performed. For descriptive comparisons, patients were divided into high- and low-RCD.score groups according to the median weighted RCD.score within the Day 1 cohort.

APACHE II scores were compared between RCD.score groups using the Wilcoxon rank-sum test. Discrimination of survivors and nonsurvivors by continuous RCD.score was assessed using ROC analysis with a 95% confidence interval for the AUC.

For the exploratory joint-stratification analysis, high APACHE II was prespecified as a score ≥20, whereas high RCD.score was defined as a weighted score above the cohort median. This generated four groups: low APACHE II/low RCD.score, low APACHE II/high RCD.score, high APACHE II/low RCD.score, and high APACHE II/high RCD.score. Mortality proportions and exact 95% binomial confidence intervals were calculated for each group. The high-APACHE II/high-RCD.score group was compared with all remaining patients using Fisher’s exact test and an exact odds ratio. A secondary comparison was performed between high- and low-RCD.score patients among those with APACHE II scores of 20 or higher.

Incremental discrimination was assessed by comparing a logistic model containing APACHE II alone with a model containing APACHE II and continuous RCD.score. AUCs were compared using DeLong’s test. Continuous net reclassification improvement and integrated discrimination improvement were calculated, with confidence intervals estimated using 2,000 nonparametric bootstrap replicates. Because of the limited sample size, the external incremental and joint-stratification analyses were considered exploratory.

**Per-cell and sample-level RCD.score analyses in GSE167363**

The complete GSE167363 dataset comprised 12 samples from seven individuals, including paired baseline and 6-h samples from five patients with sepsis and two healthy control samples. After quality-control filtering, 64,244 cells were retained for the cell-level mapping analysis. Standard preprocessing included log-normalization, highly variable gene identification, scaling, principal component analysis, UMAP embedding, and graph-based clustering. Cell types were annotated using SingleR with the Monaco Immune reference.

For each cell, z-score-standardized expression values of SMC4, SLC5A6, POLR3GL, MAD2L2, EXOC7, and COG1 were multiplied by their fixed coefficients and summed to obtain the per-cell RCD.score. Cells with an RCD.score above the median across all retained cells were classified as high-RCD.score cells. For each annotated cell population, enrichment of high-RCD.score cells were evaluated by comparing that population with all other cells using Fisher’s exact test, followed by Benjamini–Hochberg correction.

The additional sample-level analysis was restricted to the two healthy controls and five baseline sepsis samples, excluding the paired 6-h samples to avoid repeated observations from the same patients. Major immune-cell compartments were defined as B cells, T cells, natural killer cells, and monocytes. The T-cell compartment combined cells annotated as CD4-positive T cells, CD8-positive T cells, or T cells.

A cell type was retained only when all seven biological samples contained at least 100 cells of that type. All four major compartments satisfied this criterion, yielding 28 sample-by-cell-type profiles and 31,187 cells in total. The minimum number of cells in an included sample-by-cell-type profile was 123.

The six-gene score was calculated from log-normalized single-cell expression using the fixed bulk-derived coefficients. Within each biological sample and major cell compartment, the mean cellular RCD.score was summarized. The weighted contribution of an individual model gene was defined as its fixed coefficient multiplied by its mean normalized expression within the corresponding sample and cell type. For monocytes, sepsis-associated contribution differences were calculated by subtracting the mean contribution in healthy controls from that in baseline sepsis samples. Given that only two healthy control samples were available, sample-level score comparisons were treated as descriptive.

**Cell-type-specific pseudobulk differential-expression analysis**

Raw RNA counts from the two healthy control samples and five baseline sepsis samples were aggregated separately for each biological sample and major immune-cell compartment. This generated seven pseudobulk profiles for each of the four cell types and 28 pseudobulk profiles in total. Biological samples, rather than individual cells, were treated as the units of replication.

Pseudobulk analyses were conducted separately for B cells, T cells, natural killer cells, and monocytes using edgeR. For each cell type, a DGEList object was constructed from the aggregated raw-count matrix. Lowly expressed genes were removed using filterByExpr with the corresponding design matrix. Library sizes were recalculated after filtering, and between-sample normalization was performed using the trimmed mean of M values method.

The design matrix included healthy control and baseline sepsis groups, with healthy controls used as the reference. Dispersion was estimated using estimateDisp with robust estimation. Robust quasi-likelihood generalized linear models were fitted using glmQLFit, and the baseline-sepsis-versus-control contrast was tested using glmQLFTest.

Benjamini–Hochberg-adjusted FDR values below 0.05 were used to define differentially expressed genes in the primary pseudobulk analyses, without an additional fold-change threshold. Positive log₂ fold-change values indicated higher expression in baseline sepsis samples than in healthy controls. Complete cell-type-specific pseudobulk differential-expression results are provided in Supplementary Table S19.
