# Supplementary material for: A high-risk sepsis subtype identified by regulated cell death signatures and MAD2L2 validation
Source: Front Immunol. 2026 Aug 27;17:1846661. doi: 10.3389/fimmu.2026.1846661 (PMC13557957; doi:10.3389/fimmu.2026.1846661)
Supplement: Supplementary file 3 [file Table1.docx]

1. Immunoblot images for validation of si-MAD2L2 (Supplementary Figure 4)

2. Fig. 6c

3. Fig. 6d

4. Fig. 6i
